# Protein and Signaling Pathway Responses to rhIL-6 Intervention Before Lobaplatin Treatment in Osteosarcoma Cells

**DOI:** 10.3389/fonc.2021.602712

**Published:** 2021-03-09

**Authors:** Huan Wang, Bin Li, Kang Yan, Yonghong Wu, Yanhua Wen, Yunyan Liu, Pei Fan, Qiong Ma

**Affiliations:** ^1^Orthopedic Oncology Institute, Department of Orthopedic Surgery, Tangdu Hospital, Fourth Military Medical University, Xi'an, China; ^2^Department of Orthopedics, The Second Affiliated Hospital of Wenzhou Medical University, Yuying Children's Hospital, Wenzhou, China

**Keywords:** osteosarcoma, rhIL-6 intervention, lobaplatin, liquid chromatography-tandem mass spectrometry, protein–protein interaction, FUBP1

## Abstract

Lobaplatin is a third-generation platinum-based antineoplastic agent and is widely used for osteosarcoma treatment before and after tumor removal. However, treatment failure often results from lobaplatin drug resistance. In our study, we found that SaOS-2 and SOSP-9607 osteosarcoma cells became less sensitive to lobaplatin after treatment with exogenous interleukin (IL)-6. Quantitative proteomic analysis was performed to elucidate the underlying mechanism in SaOS-2 osteosarcoma cells. Cells were divided into a control group (CG), a lobaplatin treatment group (LG), a recombinant human IL-6 (rhIL-6), and a lobaplatin treatment group (rhILG). We performed three biological replicates in each group to compare the differential protein expression between groups using a tandem mass tag (TMT) labeling technology based on liquid chromatography-tandem mass spectrometry (LC-MS/MS). A total of 1,313 proteins with significant differential expression was identified and quantified. The general characteristics of the significantly enriched proteins were identified by Gene Ontology (GO) and Kyoto Encyclopedia of Genes and Genomes (KEGG) enrichment analyses, and protein–protein interaction (PPI) analysis was conducted using IntAct and STRING. In total, 31 proteins were further verified by parallel reaction monitoring (PRM), among which ras GTPase-activating protein-binding protein 1 (G3BP1), fragile X mental retardation syndrome-related protein 1 (hFXR1p), and far upstream element-binding protein 1 (FUBP1) were significantly differentially expressed. Immunohistochemistry results showed that these three proteins are highly expressed in specimens from platinum-resistant osteosarcoma patients, while the proteins are negatively or weakly expressed in specimens from platinum-sensitive osteosarcoma patients. The immunofluorescence staining results were in accord with the immunohistochemistry staining results. siRNA knockdown of FUBP1 showed a strikingly decreased IC50 value for lobaplatin in FUBP1-silenced cells, which verified the role of FUBP1 in the drug susceptibility of osteosarcoma and the potential therapeutic value for increasing the sensitivity to lobaplatin. This is the first proteomic study on a rhIL-6 intervention before lobaplatin treatment in osteosarcoma cells.

## Introduction

Osteosarcoma accounts for approximately 44.6% of malignant bone tumors and is the most common primary malignant bone tumor clinically. Osteosarcoma usually occurs in children and young adolescents and demonstrates poor prognosis (1, 2). As osteosarcoma is considered less sensitive to radiotherapy, chemotherapy has been the traditional treatment accompanying surgery to improve the survival of tumor-suffering patients (3). Platinum-based drugs, such as cisplatin, carboplatin, and lobaplatin, are extensively used for osteosarcoma patients (4, 5). Lobaplatin is a third-generation platinum-based chemotherapy drug with ideal antitumor activity and stability. However, osteosarcoma becomes less sensitive to lobaplatin after one or two courses of treatment.

Interleukin (IL)-6 is a pleiotropic cytokine that can act as a resistance factor against some antitumor drugs, such as doxorubicin, VP-16, and cisplatin (6). It has been reported that elevated IL-6 is associated with neoplastic growth and apoptosis (7–9). However, the underlying comprehensive mechanism of IL-6 treatment on the decreased sensitivity to platinum-based chemotherapeutics in osteosarcoma cells has not yet been systemically elucidated.

Proteomic analysis has shown great strength for large-scale protein investigations to explain the specific mechanism of the role of exogenous IL-6 in drug treatment for osteosarcoma. However, there are no studies on the proteomic response to IL-6 usage before lobaplatin treatment at the protein level. In this study, we investigated the differential protein expression after recombinant human IL-6 (rhIL-6) intervention before lobaplatin treatment of an osteosarcoma SaOS-2 cell model and revealed potential biomarkers that indicate the decreased sensitivity of osteosarcoma to lobaplatin.

## Materials and Methods

### Cell Culture and Treatment

The human osteosarcoma cell lines SaOS-2 and SOSP-9607 were maintained in our laboratory (10) and grown in Dulbecco's modified Eagle's medium and RPMI 1640 medium (HyClone, USA) with 10% fetal bovine serum (Gibco, USA). Cells were cultured at 37°C in a humidified atmosphere with 5% CO_2_. Recombinant human IL-6 (PeproTech, USA) was used to pretreat tumor cells before lobaplatin treatment.

### Cell Viability Assay

Cells were seeded in a 96-well plate at 6 × 10^3^ cells per well and divided into the following four groups: CG (cells treated with the solvent of rhIL-6 and the solvent of lobaplatin), rhIL-6G (cells treated with 60 ng/mL rhIL-6 and the solvent of lobaplatin), LG (cells treated with the solvent of rhIL-6 and 10 μg/mL lobaplatin 8 h later), and rhILG (cells treated with 60 ng/mL rhIL-6 and 10 μg/mL lobaplatin 8 h later). Each group had three replicates. CCK-8 assay was then performed after 24 h of lobaplatin treatment according to the manufacturer's instructions (Dojindo, Japan), and the absorbance at 450 nm was measured to detect the cell viability with a multiscan reader (Thermo Scientific, USA). The results are expressed as the mean optical density ± SEM (*n* = 3).

### Apoptosis Assessment

Apoptotic cells were measured using an annexin V-FITC/PI detection kit (BD Biosciences, USA) and analyzed by flow cytometry (Beckman Coulter, USA) according to the manufacturer's instructions. A total of 3 × 10^5^ cells were seeded in a 6-well plate, the treatment was the same as described previously, and each group contained three replicates. Cells were then collected by gentle digestion with trypsin, washed with precooled PBS, and resuspended in 400 μL of binding buffer. Five microliters of annexin V-FITC and 1 μg/mL PI were used to stain cells in the CG, rhIL-6G, LG, and rhILG for 15 min in the dark and then analyzed immediately. The early apoptotic cells were annexin V+/PI–, and the late apoptotic cells were annexin V+/PI+.

The cell nuclei were stained with Hoechst 33342 solution (Roche, Switzerland). Cells were seeded in a 24-well plate at 7 × 10^4^ cells per well and divided into the CG, rhIL-6G, LG, and rhILG. The treatment was the same as described previously, and each group contained three replicates. The cultures were stained in the incubator for 15 min at 37°C at a concentration of 5 μmol/ml. After incubation, the cells were washed with PBS three times, and the fluorescence was detected with a fluorescence microscope (Olympus, Japan).

### Proteomics Analysis

#### Sample Preparation

A total of 8 × 10^6^ cells were seeded in nine 15-cm culture dishes, and each group contained three dishes for replicates. After the treatment as previously described, cells were washed three times and collected by scraping in precooled PBS on ice. Cell pellets were centrifuged at 4°C, and the supernatants were removed. Samples were then rapidly frozen in liquid nitrogen for 30 s. Cell pellets were lysed with SDT buffer (4% SDS and 100 mM Tris-HCl at pH 7.6), boiled for 15 min and centrifuged at 14,000 × g for 40 min. The supernatants were collected and quantified with the BCA Protein Assay Kit (Pierce, USA). Samples were stored at −80°C until use. A total of 20 μg of proteins for each sample was mixed with 5X loading buffer and boiled for 5 min. The proteins were separated on a 12.5% SDS-PAGE gel. Protein bands were visualized by Coomassie Blue R-250 staining.

For the filter-aided sample preparation (FASP) and digestion (11), 200 μg of proteins from each sample was incorporated into 30 μL SDT buffer (4% SDS, 100 mM DTT, and 150 mM Tris-HCl at pH 8.0). DTT and other low-molecular-weight components were removed with uric acid (UA) buffer (8 M urea and 150 mM Tris-HCl at pH 8.5) by repeated ultrafiltration (Sartorius, 30 kDa). Then, 100 μL of 100 mM iodoacetamide (IAA) in UA buffer was added to block reduced cysteine residues, and the samples were incubated for 30 min under darkness. The filters were washed three times with 100 μL UA buffer and then twice with 100 μL of 0.1 M tetraethyl-ammonium bromide (TEAB) buffer. Finally, the protein suspensions were digested with 4 μg trypsin (Promega, USA) in 40 μL 0.1 M TEAB buffer overnight at 37°C, and the resulting peptides were collected. By calculating the frequency of tryptophan and tyrosine in vertebrate proteins, an extinction coefficient of 1.1 of 0.1% (g/L) solution was chosen to estimate the peptide concentration using a UV spectrometer at 280 nm.

A 100 μg peptide mixture of each sample was labeled using the tandem mass tag (TMT) reagent according to the manufacturer's instructions (Thermo Fisher Scientific, USA). The peptides in the CG, LG, and rhILG were labeled with TMT-126, TMT-127N, TMT-127C, TMT-128N, TMT-128C, TMT-129N, TMT-129C, TMT-130C, and TMT-131, with three biological replicates. TMT-labeled peptides were fractionated by reversed-phase (RP) chromatography using an Agilent 1260 Infinity II HPLC. The peptide mixture was diluted with buffer A [10 mM HCOONH_4_ and 5% acetonitrile (ACN) at pH 10.0] and loaded onto an XBridge Peptide BEH C18 Column, 130 Å, 5 μm, 4.6 × 100 mm column. The peptides were eluted at a flow rate of 1 ml/min with a gradient of 0–7% buffer B (10 mM HCOONH_4_ and 85% ACN at pH 10.0) for 5 min, 7–40% buffer B for 5–40 min, 40–100% buffer B for 45–50 min, and 100% buffer B for 50–65 min. The elution was monitored by the absorbance at 214 nm, and fractions were collected every 1 min for 50 min. The collected fractions were dried down via vacuum centrifugation.

#### MS Analysis

Each fraction was injected for nanoliquid chromatography-tandem mass spectrometry (nanoLC-MS/MS) analysis. The peptide mixture was loaded onto a C18 RP analytical column (Thermo Fisher Scientific, Acclaim PepMap RSLC 50 μm × 15 cm, nanoViper, P/N164943) in buffer A (0.1% formic acid) and separated with a 1.5-h linear gradient of buffer B (80% ACN and 0.1% formic acid) at a flow rate of 300 nl/min. Specifically, the peptides were eluted as follows: 6% buffer B for 5 min, 6–28% buffer B for 63 min, 28–38% buffer B for 10 min, 38–100% buffer B for 7 min, and 100% buffer B for 5 min. Liquid chromatography-tandem mass spectrometry analysis was performed on a Q Exactive Plus mass spectrometer (Thermo Fisher Scientific, USA) that was coupled to an Easy nLC (Thermo Fisher Scientific, USA) for 90 min. The mass spectrometer was operated in the positive ion mode. MS data were acquired using a data-dependent top 10 method dynamically choosing the most abundant precursor ions from the survey scan (350–1,800 m/z) for higher energy collisional dissociation (HCD) fragmentation. The automatic gain control (AGC) target was set to 3.0E6, and the maximum injection time was 45 ms. Survey scans were acquired at a resolution of 70,000 at 200 m/z, the resolution for the HCD spectra was set to 17,500 at 200 m/z, and the isolation width was 2 m/z. The normalized collision energy (NCE) was 30 eV.

MS/MS spectra were searched using the MASCOT engine (Matrix Science, UK; version 2.6, RRID: SCR_014322) embedded in Proteome Discoverer 2.1. The principal parameters were set as follows: peptide false discovery rate (FDR) ≤ 0.01 and Fragment Mass Tolerance of 0.1 Da. Proteins with a fold change>1.2 or <0.83 and *p* < 0.05 (Student's *t*-test) were considered to be differentially expressed proteins.

### GO and KEGG Pathway Analyses

All protein sequences were aligned to the Homo sapiens database that was downloaded from NCBI (ncbi-blast-2.2.28+-win32.exe), and only the sequences in the top 10 with an *E*-value <1E-3 were retained. Then, the GO terms (database version: go_201910.obo, RRID: SCR_002811) of the sequences with the top bit score were selected by Blast2GO, and the annotations of GO terms to proteins were completed by the Blast2GO Command Line. After the elementary annotation, InterProScan was used to search the EBI database by motif, and the functional information for each motif was added to the proteins to improve the annotation. Then, further improvements to the annotation and connections between GO terms were carried out by ANNEX. Fisher's exact test was used to enrich GO terms by comparing the number of differentially expressed proteins and total proteins correlated to the GO terms. Pathway analysis was performed using the KEGG database (database version: KO_INFO_END_20191021, RRID: SCR_012773). Fisher's exact test was used to identify the significantly enriched pathways by comparing the number of differentially expressed proteins and total proteins correlated to pathways.

#### Cluster Analysis

Cluster analysis was performed using STEM (12) to ensure the changing trends of various kinds of proteins and proteins with similarly changing trends were classified in a cluster accordingly. Significance analysis was performed for each cluster, and clusters with statistical significance were acquired.

#### PPI Analysis

The IntAct molecular interaction database (database version: IntAct View 4.2.16, SCR_006944) was utilized to study the relationship between the differentially expressed proteins using their distinct gene symbols, and the Cytoscape software was used to visualize the functional PPI networks. Protein–protein interaction (PPI) information was also retrieved from the STRING database, which shows direct experimental interactions and predicted interactions using computational algorithms. In both of the above databases, the degrees of connectivity for each differentially expressed protein between the LG and rhILG were calculated to evaluate the importance of proteins in the PPI network. The intersection proteins of the two databases were considered to be the target proteins.

### PRM Validation

Parallel reaction monitoring (PRM) validation was performed among the differentially expressed proteins to verify the proteomic analysis based on the TMT label-based LC-MS/MS. In total, 31 proteins were analyzed, including three proteins of interest, namely, ras GTPase-activating protein-binding protein 1 (G3BP1), far upstream element-binding protein 1 (FUBP1), and fragile X mental retardation syndrome-related protein 1 (hFXR1p). Two micrograms of peptide mixture was loaded onto the C18 RP analytical column (Thermo Fisher Scientific, Acclaim PepMap RSLC 50 μm × 15 cm, nanoViper, P/N164943) in buffer A (0.1% formic acid) and separated with a non-linear gradient of buffer B (80% ACN and 0.1% formic acid) at a flow rate of 300 nl/min. The gradient was 2–8% buffer B for 1 min, 8–28% buffer B for 45 min, 28–40% buffer B for 50 min, 40–90% buffer B for 56 min, and 90% buffer B for 3 min.

Peptide fragmentation and targeted PRM MS were performed using a Q Exactive Mass Spectrometer (Thermo Scientific, USA). The mass spectrometer was operated in the positive ion mode. MS data were acquired with the following settings: (1) Full-MS: scan range (m/z) = 350–1,500; resolution = 60,000; AGC target = 1E6; and maximum injection time = 50 ms; (2) PRM: resolution = 17,500; AGC target = 1E5; maximum injection time = 50 ms; isolation window = 2 m/z; and NCE = 27%. The MS RAW file was converted to the mzXML format via the MSConvertGUI software. The resulting mass spectrum mzXML file was analyzed using the Skyline 3.6 software for PRM data.

Proteome Discoverer (v. 2.1) and Skyline (v. 3.6) were used for raw MS data processing. The MASCOT engine was applied to search the derived peak list according to the UniProt Homo sapiens protein database in Proteome Discoverer. The peptides were generated using trypsin as the enzyme. The precursor mass tolerance was specified as 10 ppm, and for MS2 fragments, the tolerance was 0.05 Da. Carbamidomethyl was chosen as the fixed modification, while acetyl and oxidation were set as the variable modifications. A reverse database search strategy was used with the peptide and protein FDR set to 1%. The default settings were applied to all other operations. A spectral library with a cut-off score of 0.99 was built with the MS/MS table file output by MASCOT in Skyline.

### Clinical Specimens, Immunohistochemical, and Immunofluorescence Staining

Specimens were collected from 30 osteosarcoma patients who underwent platinum-based chemotherapy and tumor resection at Tangdu Hospital, Fourth Military Medical University between January 2008 and December 2018. Tumors with regression of more than 50% were categorized into the chemotherapy-sensitive group according to a previous study that evaluated the chemotherapy response in rectal cancer (13). In total, 15 patients were categorized into the chemotherapy-sensitive group, and the rest were categorized into the chemotherapy-resistant group. Studies concerning patients and their specimens were approved by the Ethics Committee of the Fourth Military Medical University, and written informed consent was provided. Formalin-fixed paraffin-embedded specimens were then sectioned into 4–6-μm sections (LEICA, Germany), affixed onto microscope slides, and heated in an oven at 60°C overnight. The following procedures were performed according to the instructions provided by Abcam Company (Abcam, USA). The anti-G3BP (Abcam, Cat# ab56574, RRID: AB_941699), anti-FXR1 (Abcam, Cat#ab129089, RRID: AB_11154960), and anti-FUBP1 (Abcam, Cat # ab 181111) antibodies were used at dilutions of 1:100, 1:100, and 1:250, respectively. DAB (GK347010, Gene Tech) or fluorescent secondary antibody (Affinity Biosciences, Cat# S0011, RRID: AB_2844800) was used to detect proteins expression. Two different pathologists independently scored the tissues, which were assigned based on the positive cell numbers combined with the intensity of staining as described previously (14).

### siRNAs and Transfection

Small interfering RNA (siRNA) oligonucleotides sequences were synthesized (GenePharma, China). The siRNA sequences targeting human G3BP1 are: RNAi#1, 5′-GGGAAUUUGUGAGACAGUAT T-3′ (sense), 5′-UACUGUCUCACAAAUUCCCTT-3′ (antisense); RNAi#2, 5′-GCCUGAGCCAGUAUUAGAATT-3′ (sense), 5′-U UCU AAUACUGGCUCAGGCTT-3′ (antisense); and RNAi#3, 5′-GCGAGAACAACGAAUAAAUTT-3′ (sense), 5′-AUUUAUUCGUUGUUCUCGCTT-3′ (antisense). The siRNA sequences targeting human FUBP1 are: RNAi#1, 5′-GGUGUUCGC AUUCAGUUUATT-3′ (sense), 5′-UAAACUGAAUGCGAACACCTT-3′ (antisense); RNAi#2, 5′-GGUGCUGACAAACCUCUUATT-3′ (sense), 5′ -UAA GAGGUUUGUCAGCACCTT-3′ (antisense); and RNAi#3, 5′-CGGCAACUCAUAGAAGAAATT-3′ (sense), 5′-UUUCUUCUAUG AGUUGCCGTT-3′ (antisense). The siRNA sequences targeting human FXR1 are: RNAi#1, 5′-GGAGC UGACGGUGGAGGUUTT-3′ (sense), 5′-AACCUCCACCGUCAGCUCCTT-3′ (antisense); RNAi#2, 5′-GCAAAUGACCAAGAGCCAUTT-3′ (sense), 5′-AUGGCUCUUGGUCAUUUGCTT-3′ (antisense); and RNAi #3,5′-GCU AGAGGUUUCUUGGAAUTT-3′ (sense), 5′-AUUCCAAGAAACCUCUAGCTT-3′ (antisense). A scrambled siRNA was used as the control. siRNA transfection was performed using Lipofectamine 3000 (Invitrogen, USA) according to the manufacturer's instruction.

### Statistical Analysis

Each experiment was carried out at least three times, and the mean value was calculated. Statistical analysis was performed with SPSS 25.0 (IBM, RRID: SCR_002865) and GraphPad Prism 7 software (GraphPad, RRID: SCR_002798). Data are presented as the mean ± SEM. The IC50 was calculated by regression analysis. Cell viability was obtained using one-way ANOVA. Differences between two groups were analyzed by Student's *t*-test, and *p* < 0.05 was considered statistically significant.

## Results

### Exogenous IL-6 Reduces the Susceptibility of SaOS-2 and SOSP-9607 Osteosarcoma Cells to Lobaplatin

**Figure 1:**
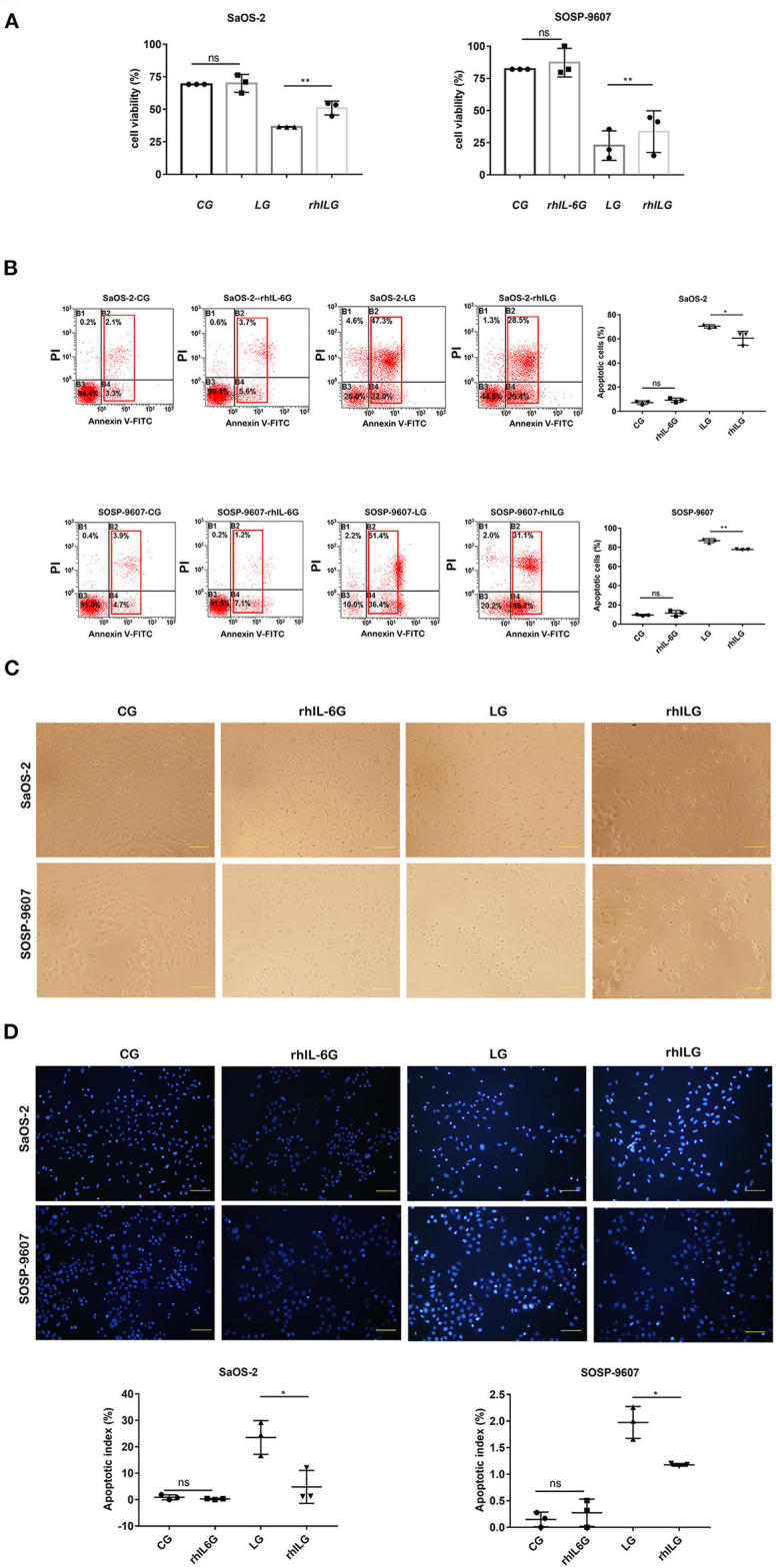
rhIL6 intervention before lobaplatin treatment of osteosarcoma cells increased cell viability and reduced SaOS-2 and SOSP-9607 cell apoptosis. **(A)** Cell viability measured by CCK-8 assay in the CG, rhIL-6G, LG, and rhILG. Experiments were performed with at least three biological replicates, and a *p* ≤ 0.05 was considered statistically significant. **(B)** Cells in each group were stained with annexin V and PI and measured by flow cytometry. Early and late apoptotic cells were demonstrated as annexin V+/PI– (early apoptosis) and annexin V+/PI+ (late apoptosis), respectively. **(C)** Morphological differences in osteosarcoma cells in the CG, rhIL-6G, LG, and rhILG were observed by bright-field microscopy (×200), scale bar = 100 μm. **(D)** Morphological differences in osteosarcoma cell nuclei were detected by Hoechst 33342 staining and observed by fluorescence microscopy (×200), scale bar = 100 μm. *p < 0.05; **p < 0.01.

To assess the potential role of IL-6 in the process of lobaplatin resistance in osteosarcoma cells, 60 ng/mL rhIL-6 was applied to SaOS-2 and SOSP-9607 osteosarcoma cells 8 h before lobaplatin treatment. After 24 h, a Cell Counting Kit-8 (CCK-8) assay was performed to detect the viability of tumor cells. It was demonstrated that the average cell viability of SaOS-2 and SOSP-9607 osteosarcoma cells treated with rhIL-6 and then with lobaplatin (rhIL-6 and lobaplatin group, rhILG) was 73.58% ± 0.021 and 40.86% ± 0.054, while that of SaOS-2 and SOSP-9607 cells treated only with lobaplatin (lobaplatin group, LG) was 52.65% ± 0.0009 and 27.66% ± 0.038, respectively, indicating that SaOS-2 and SOSP-9607 cells pretreated with rhIL-6 exhibit greater resistance to lobaplatin (*p* = 0.0093 and *p* = 0.0023, respectively). No significant difference was found between the CG and rhIL-6G (*p* = 0.087). Tumor cell apoptosis in the different groups was then detected by flow cytometry. The average proportions of apoptotic cells were 7.13% ± 0.0041, 9.10% ± 0.0049, 70.4% ± 0.0037, and 60.5% ± 0.016 in the CG, rhIL-6G, LG, and rhILG for SaOS-2 cells and 9.40% ± 0.027, 11.37% ± 0.0079, 87.03% ± 0.0056, and 77.77% ± 0.0009 in the CG, rhIL-6G, LG, and rhILG for SOSP-9607 cells, respectively, which showed that rhIL-6 treatment significantly reduced the apoptosis of SaOS-2 and SOSP-9607 cells induced by the antineoplastic agent lobaplatin (*p* < 0.05) and enhanced the resistance of tumor cells to the drug. Hoechst 33342 was used to stain osteosarcoma cell nuclei of different groups, and the cells were observed by fluorescence microscopy. Cells in the LG exhibited obvious apoptotic morphology, which showed nuclear pyknosis and asymmetric chromatin condensation compared with cells in the rhILG, CG, and rhIL-6G (Figure 1).

### Hierarchical Cluster

**Figure 2:**
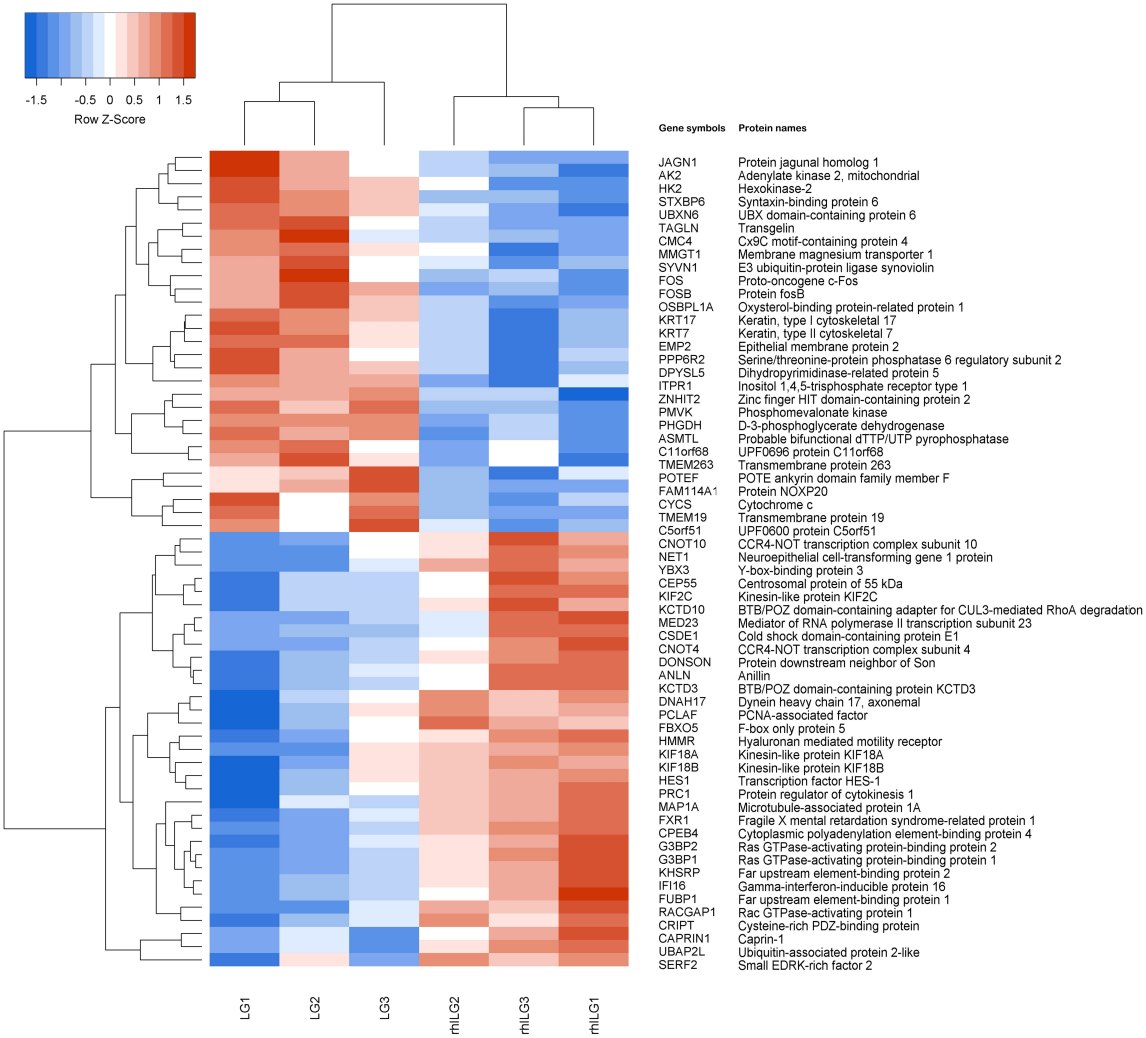
Hierarchical clustering of the differentially expressed proteins in SaOS-2 osteosarcoma cells between the LG and rhILG. Each group contains three samples. The heatmap is a visualized demonstration of the protein distribution in different samples. The red color represents upregulation, and the blue color represents downregulation. The upper dendrogram illustrates the clustering analysis of different samples in different groups, and the left dendrogram shows the clustering analysis of different proteins in different samples. A total of 62 proteins were significantly changed between the LG and rhILG, including 33 upregulated proteins and 29 downregulated proteins (*p* < 0.05).

Tandem mass tag liquid chromatography-tandem mass spectrometry was performed to identify some prominent proteins during rhIL-6 intervention before lobaplatin treatment in SaOS-2 osteosarcoma cells. A total of 1,313 differentially expressed proteins were identified and quantified, of which 62 proteins (33 upregulated; 29 downregulated) were found between the rhILG and LG (Figure 2), and 1,251 proteins (819 upregulated; 432 downregulated) were found between the LG and CG (Supplementary Figure 1). All the above proteins were either significantly up- or down-regulated by greater than a 1.2-fold change (*p* < 0.05). The heatmap of the hierarchical clustering demonstrated the differentially expressed proteins, which gives us a better visualization of the overall protein changes.

### GO and KEGG Pathway Enrichment Analyses

**Figure 3:**
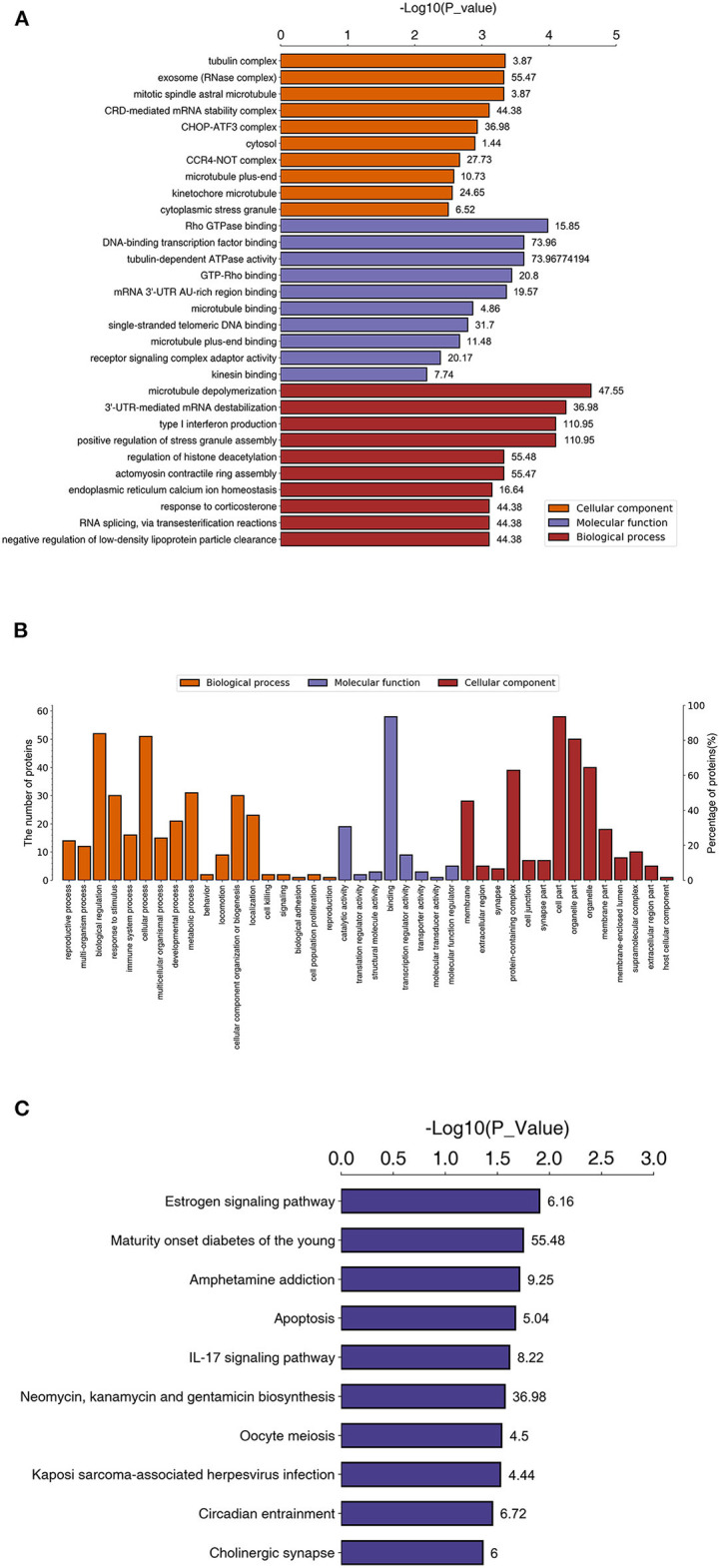
Enriched GO and KEGG pathways between proteins in the LG and rhILG. Three replicates were performed for each group. **(A)** Top 10 enriched GO terms using Fisher's exact test for the biological process (BP), molecular function (MF), and cellular component (CC) categories. The vertical axis represents GO terms in each category, and the numbers beside the bars are enrichment factors, which represent the significance and reliability of proteins enriched in this item. The reliability of the proteins in an item was enhanced when the value increased. The horizontal axis demonstrates the –log 10 (*p*-value) of each item. **(B)** Total level 2 GO enrichment in the BP, MF, and CC categories. The vertical axes represent numbers of differentially expressed proteins that belong to a specific GO item (left) and the ratio of proteins in the item to the total differentially expressed proteins. **(C)** Significantly enriched pathways between the LG and rhILG using Fisher's exact test. The horizontal axis shows the significance of each pathway in the form of –log 10 (*p*-value). Numbers beside the bars are enrichment factors of each enriched pathway.

Gene Ontology (GO) and Kyoto Encyclopedia of Genes and Genomes (KEGG) pathway analyses were performed between the rhILG and LG. The most enriched GO terms were annotated as Rho GTPase binding in the molecular function category (GO: 0017049, three proteins, enrichment factor = 15.85, *p* = 3.59165E-04), tubulin complex in the cellular compartment category (GO: 0045298, nine proteins, enrichment factor = 3.87, *p* = 4.53549E-04), and microtubule depolymerization in regard to the biological process category (GO: 0007019, three proteins, enrichment factor = 47.55, *p* = 2.37872E-05) (Figures 3A,B, Table 1).

**Table 1:** Enrichment of proteins and signaling pathways between the LG and rhILG groups based on GO and KEGG analysis.

**Terms**	**Protein number**	***p*-Value**	**FDR**	**Enrichment factor**	**Protein names (gene names)**
**GO**					
(MF) Rho GTPase binding	3	3.592E-04	0.5243	15.85	Syntaxin-binding protein 6 (STXBP6); Anillin (ANLN); BTB/POZ domain-containing adapter for CUL3-mediated RhoA degradation protein 3 (KCTD10)
(CC) Tubulin complex	9	4.535E-04	0.1962	3.87	Dihydropyrimidinase-related protein 5 (DPYSL5); Cysteine-rich PDZ-binding protein (CRIPT); Rac GTPase-activating protein 1 (RACGAP1); Fragile X mental retardation syndrome-related protein 1 (FXR1); Protein regulator of cytokinesis 1 (PRC1); Kinesin-like protein (KIF18A); Kinesin-like protein (KIF2C); Kinesin-like protein (KIF18B); Microtubule-associated protein 1A (MAP1A)
(BP) Microtubule depolymerization	3	2.379E-05	1	47.55	Kinesin-like protein (KIF18A); Kinesin-like protein (KIF2C); Kinesin-like protein (KIF18B)
**KEGG PATHWAYS**					
Estrogen signaling pathway	3	0.01248	0.4391	6.16	Proto-oncogene (FOS); Keratin; type I cytoskeletal 17 (KRT17); Inositol 1,4,5-trisphosphate receptor type 1 (ITPR1)
Apoptosis	3	0.02134	0.4391	5.04	Proto-oncogene (FOS); Cytochrome c (CYCS); Inositol 1,4,5-trisphosphate receptor type 1 (ITPR1)
Amphetamine addiction	2	0.01942	0.4391	9.24	Protein fosB (FOSB); Proto-oncogene (FOS)
IL-17 signaling pathway	2	0.02428	0.4926	8.22	Protein fosB (FOSB); Proto-oncogene (FOS)
Oocyte meiosis	3	0.02874	0.6082	4.50	Inositol 1,4,5-trisphosphate receptor type 1 (ITPR1); Cytoplasmic polyadenylation element-binding protein 4 (CPEB4); F-box only protein 5 (FBXO5)
Kaposi sarcoma-associated herpesvirus infection	3	0.02975	0.4961	4.44	Proto-oncogene (FOS); Cytochrome c (CYCS); Inositol 1,4,5-trisphosphate receptor type 1 (ITPR1)
Circadian entrainment	2	0.03528	0.4391	6.72	Proto-oncogene (FOS); Inositol 1,4,5-trisphosphate receptor type 1 (ITPR1)
Cholinergic synapse	2	0.04350	0.4391	6.00	Proto-oncogene (FOS); Inositol 1,4,5-trisphosphate receptor type 1 (ITPR1)

*GO, gene ontology; KEGG pathways, Kyoto encyclopedia of genes and genomes pathways; MF, molecular functions; CC, cellular components; BP, biological processes; FDR, false discovery rate. GO and KEGG pathway enrichment were analyzed by the Fisher' exact test, and p < 0.05 were considered significant*.

Kyoto Encyclopedia of Genes and Genomes pathway analysis demonstrated that the estrogen signaling pathway (three proteins, enrichment factor = 6.16, *p* = 0.01248), apoptosis pathway (three proteins, enrichment factor = 5.04, *p* = 0.02134), amphetamine addiction (two proteins, enrichment factor = 9.24, *p* = 0.01942), IL-17 signaling pathway (two proteins, enrichment factor = 8.22, *p* = 0.02428), oocyte meiosis (three proteins, enrichment factor = 4.50, *p* = 0.02874), Kaposi's sarcoma-associated herpesvirus infection (three proteins, enrichment factor = 4.44, *p* = 0.02975), circadian entrainment (two proteins, enrichment factor = 6.72, *p* = 0.03528), and cholinergic synapse (two proteins, enrichment factor = 6.00, *p* = 0.04350) were significantly enriched (Figure 3C, Table 1).

### Cluster Analysis

**Figure 4:**
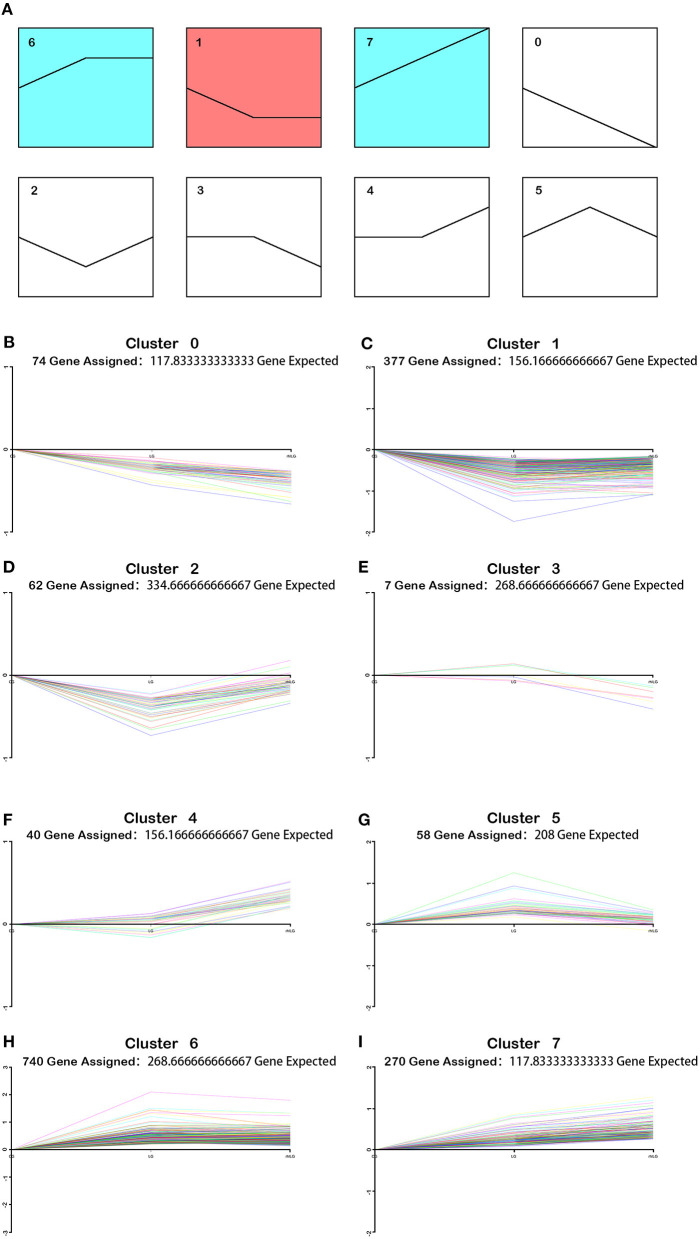
Varied trends of differentially expressed proteins between the rhILG and LG using Short Time-series Expression Miner (STEM). **(A)** Proteins were grouped into eight clusters from 0 to 7. Clusters of similar colors represent similar trends. **(B–I)** Specific trends of different clusters; each line represents a different protein. **(B)** Seventy-four proteins were contained in cluster 0, in which proteins decreased in the LG compared with in the CG and decreased in the rhILG compared with the LG. **(C)** A total of 377 proteins were contained in cluster 1, in which proteins decreased in the LG compared with in the CG and slightly increased in the rhILG compared with in the LG. **(D)** Sixty-two proteins were contained in cluster 2, in which proteins decreased dramatically in the LG compared with in the CG and increased sharply in the rhILG compared with in the LG. **(E)** Seven proteins are contained in cluster 3, in which proteins increase or decrease slightly in the LG compared with in the CG and decrease sharply in the rhILG compared with in the LG. **(F)** Forty proteins were contained in cluster 4, in which proteins increase or decrease slightly in the LG compared with the CG and increase sharply in the rhILG compared with in the LG. **(G)** Fifty-eight proteins were contained in cluster 5, in which proteins increased sharply in the LG compared with in the CG and decreased dramatically in the rhILG compared with in the LG. **(H)** A total of 740 proteins were contained in cluster 6, in which proteins increased sharply in the LG compared with in the CG and showed no obvious changes in the rhILG compared with in the LG. **(I)** A total of 270 proteins were contained in cluster 7, in which proteins increased in the LG compared with in the CG and increased in the rhILG compared with in the LG.

To understand the role of rhIL-6 pretreatment on lobaplatin treatment of SaOS-2 osteosarcoma cells, the changing trends of differentially expressed proteins between the rhILG and LG were analyzed using Short Time-series Expression Miner (STEM) and divided into eight groups. The results showed that proteins in cluster 2 decreased sharply in the LG and increased in the rhILG, while proteins in cluster 5 increased in the LG and decreased in the rhILG. Proteins in these two groups showed that rhIL-6 exhibited the opposite effect compared to lobaplatin on SaOS-2 osteosarcoma cells (Figure 4). The proteins in the above two groups with a significance of lower than 0.05 are listed in Supplementary Table 1.

### PPI Analysis

**Figure 5:**
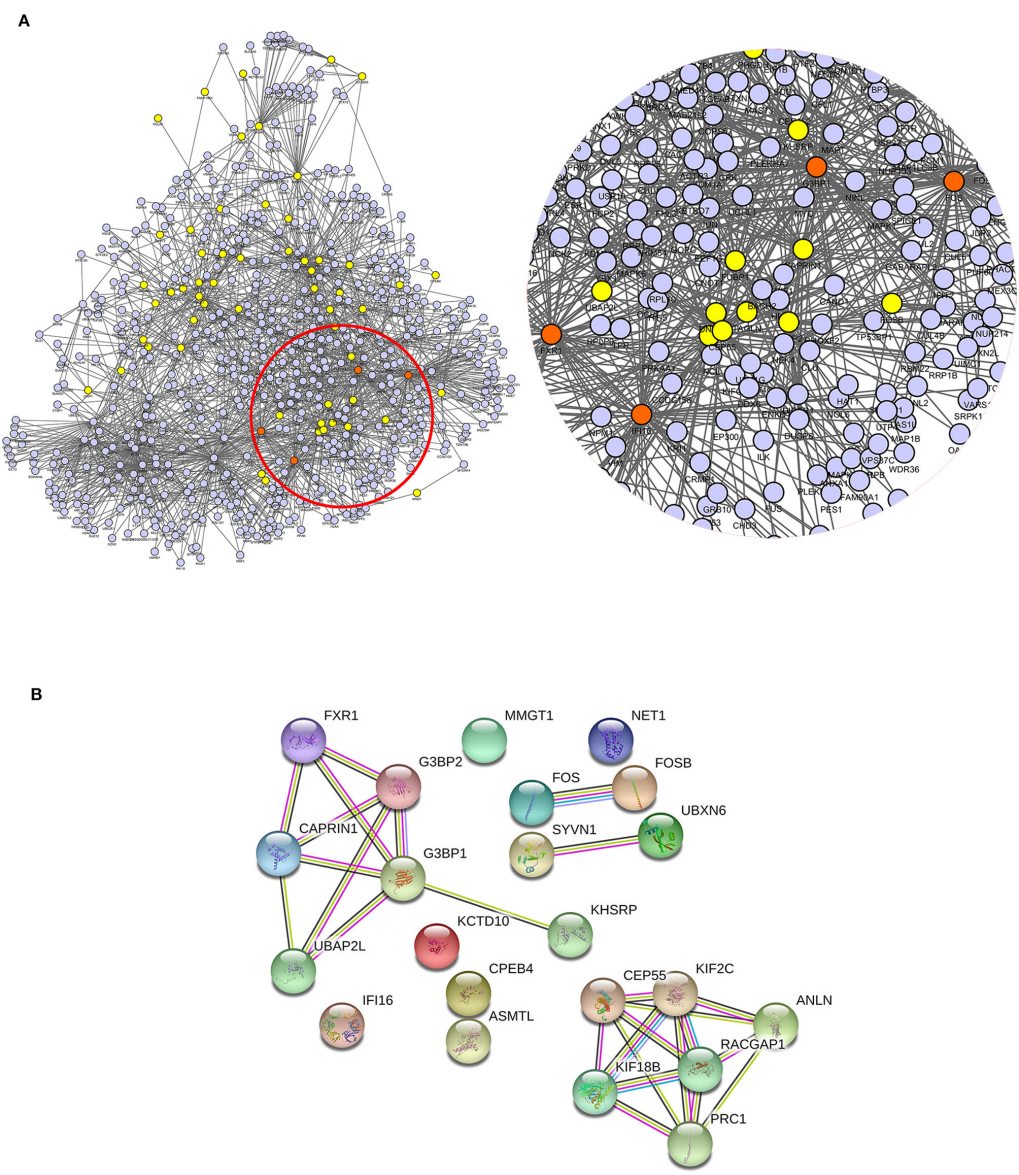
Relationship of differentially expressed proteins in clusters 2 and 5 between the LG and rhILG. **(A)** PPI network analysis was performed using the IntAct database, which shows the direct interactions among the proteins as well as the relationship between these proteins and the reported linkers. The four most enriched node proteins with a high connecting degree are shown in orange. **(B)** The PPI network was constructed using the STRING database. Blue lines represent interactions from the curated database; purple lines represent interactions that were experimentally determined; dark green lines illustrate the gene neighborhood; light green lines represent text mining interactions; red lines exhibit gene fusions; and light violet lines represent protein homology. The two databases both show G3BP1 and hFXR1p as high connectivity proteins.

Protein–protein interaction (PPI) network analysis was performed to demonstrate the relationships of the differentially expressed proteins in cluster 2 and cluster 5 using both IntAct (http://www.ebi.ac.uk/intact/main.xhtml) and STRING (http://string-db.org/cgi/input.pl). As illustrated in IntAct, the most enriched highly connective proteins between the rhILG and LG were as follows: G3BP1 (UniProtKB-Q13283), proto-oncogene (UniProtKB-P01100), and gamma-interferon-inducible protein 16 (UniProtKB-Q16666), which have interactions with three significantly differentially expressed proteins and hFXR1p (UniProtKB-P51114), which interacts with two significantly differentially expressed proteins (Figure 5A, Table 2). Meanwhile, STRING suggested that the most enriched highly connective proteins between the rhILG and LG were as follows: G3BP1 (UniProtKB-Q13283), rac GTPase-activating protein 1 (UniProtKB-Q9H0H5), and protein regulator of cytokinesis 1 (UniProtKB-O43663), which each interact with five proteins; kinesin-like protein KIF2C (UniProtKB-Q99661) and ras GTPase-activating protein-binding protein 2 (UniProtKB-Q9UN86), which each interact with four proteins; and hFXR1p (UniProtKB-P51114), kinesin-like protein KIF18B (UniProtKB-Q86Y91), caprin-1 (UniProtKB-Q14444), and centrosomal protein of 55 kDa (UniProtKB-Q53EZ4), which interact with three significantly differentially expressed proteins in the rhILG and LG (Figure 5B, Table 2). Interestingly, we found that G3BP1 and hFXR1p exhibited a high connective degree in the above different enrichment methods.

**Table 2:** Comparison of nodes and highly connected proteins in the PPI in two databases.

**Node proteins**	**Uniprot ID**	**Protein name**	**Gene name**
**IntAct**			
Ras GTPase-activating protein-binding protein 1 (three connected)	Q16666	Gamma-interferon-inducible protein 16	IFI16
	Q14444	Caprin-1	CAPRIN1
	Q9UN86	Ras GTPase-activating protein-binding protein 2	G3BP2
Gamma-interferon-inducible protein 16 (three connected)	Q13283	Ras GTPase-activating protein-binding protein 1	G3BP1
	Q14444	Caprin-1	CAPRIN1
	Q16666	Gamma-interferon-inducible protein 16	IFI16
Proto-oncogene c-fos (three connected)	Q92945	Far upstream element-binding protein 2	KHSRP
	P53539	Protein fosB	FOSB
	P01100	Proto-oncogene c-Fos	FOS
Fragile X mental retardation syndrome-related protein 1 (two connected)	Q14157	Ubiquitin-associated protein 2-like	UBAP2L
	P51114	Fragile X mental retardation syndroame-related protein 1	FXR1
**STRING**			
Ras GTPase-activating protein-binding protein 1 (five connected)	P51114	Fragile X mental retardation syndrome-related protein 1	FXR1
	Q14444	Caprin-1	CAPRIN1
	Q9UN86	Ras GTPase-activating protein-binding protein 2	G3BP2
	Q14157	Ubiquitin-associated protein 2-like	UBAP2L
	Q92945	Far upstream element-binding protein 2	KHSRP
Rac GTPase-activating protein 1 (five connected)	Q53EZ4	Centrosomal protein of 55 kDa	CEP55
	Q99661	Kinesin-like protein KIF2C	KIF2C
	Q86Y91	Kinesin-like protein KIF18B	KIF18B
	Q9NQW6	Anillin	ANLN
	O43663	Protein regulator of cytokinesis 1	PRC1
Protein regulator of cytokinesis 1 (five connected)	Q9NQW6	Anillin	ANLN
	Q9H0H5	Rac GTPase-activating protein 1	RACGAP1
	Q99661	Kinesin-like protein KIF2C	KIF2C
	Q86Y91	Kinesin-like protein KIF18B	KIF18B
	Q53EZ4	Centrosomal protein of 55 kDa	CEP55
Kinesin-like protein KIF2C (four connected)	Q53EZ4	Centrosomal protein of 55 kDa	CEP55
	Q9NQW6	Anillin	ANLN
	Q86Y91	Kinesin-like protein KIF18B	KIF18B
	Q9H0H5	Rac GTPase-activating protein 1	RACGAP1
Ras GTPase-activating protein-binding protein 2 (four connected)	P51114	Fragile X mental retardation syndrome-related protein 1	FXR1
	Q13283	Ras GTPase-activating protein-binding protein 1	G3BP1
	Q14444	Caprin-1	CAPRIN1
	Q14157	Ubiquitin-associated protein 2-like	UBAP2L
Fragile X mental retardation syndrome—related protein 1 (three connected)	Q13283	Ras GTPase-activating protein-binding protein 1	G3BP1
	Q9UN86	Ras GTPase-activating protein-binding protein 2	G3BP2
	Q14444	Caprin-1	CAPRIN1
Kinesin-like protein KIF18B (three connected)	Q53EZ4	Centrosomal protein of 55 kDa	CEP55
	O43663	Protein regulator of cytokinesis 1	PRC1
	Q9H0H5	Rac GTPase-activating protein 1	RACGAP1
Centrosomal protein of 55 kDa (three connected)	Q99661	Kinesin-like protein KIF2C	KIF2C
	Q86Y91	Kinesin-like protein KIF18B	KIF18B
	Q9H0H5	Rac GTPase-activating protein 1	RACGAP1

### PRM Validation of TMT-Based Results

**Figure 6:**
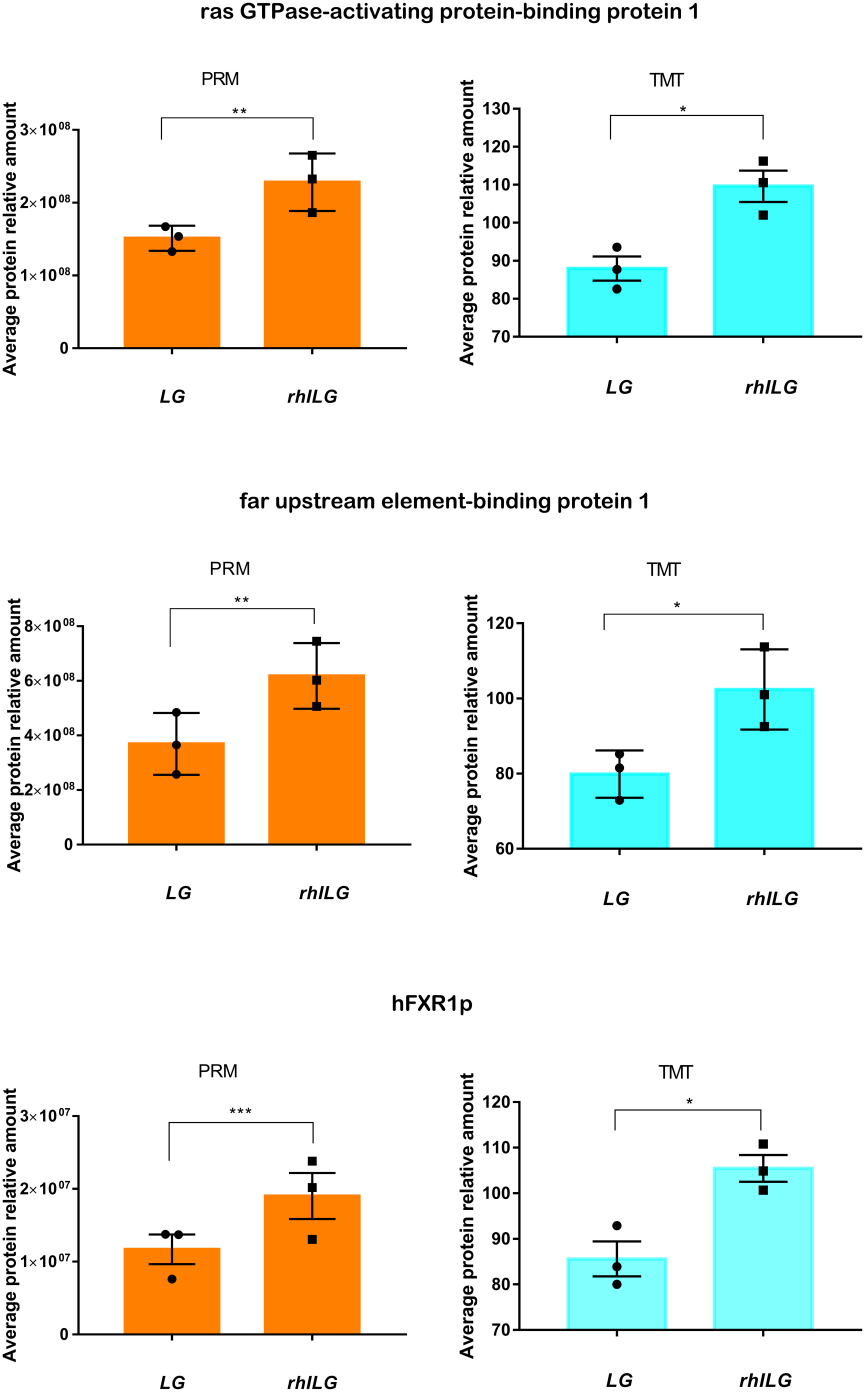
Expression of three candidate proteins by TMT label-based LC-MS/MS and PRM validation. Each group contains three samples. Student's *t*-test. *p < 0.05; **p < 0.01; ***p < 0.001.

Thirty-one differentially expressed proteins between the rhILG and LG, which were analyzed by GO and KEGG analyses and mainly in clusters 2 and 5, were selected for further verification using PRM quantitative analysis. The relative quantitative expression of each selected protein showed that 22 proteins exhibited similar trends as observed in the TMT results, while nine proteins did not demonstrate significant changes. As shown in Figure 6, the fold changes of G3BP1, FUBP1, and hFXR1p were verified to be significant in both the TMT and PRM analyses (Figure 6). However, the changes in nine proteins, including cold shock domain-containing protein E1, Rac GTPase-activating protein 1, and Kinesin-like protein, did not show statistical significance, as demonstrated by the TMT results (Supplementary Table 2).

### Immunohistochemical and Immunofluorescence Staining of Clinical Specimens

**Figure 7:**
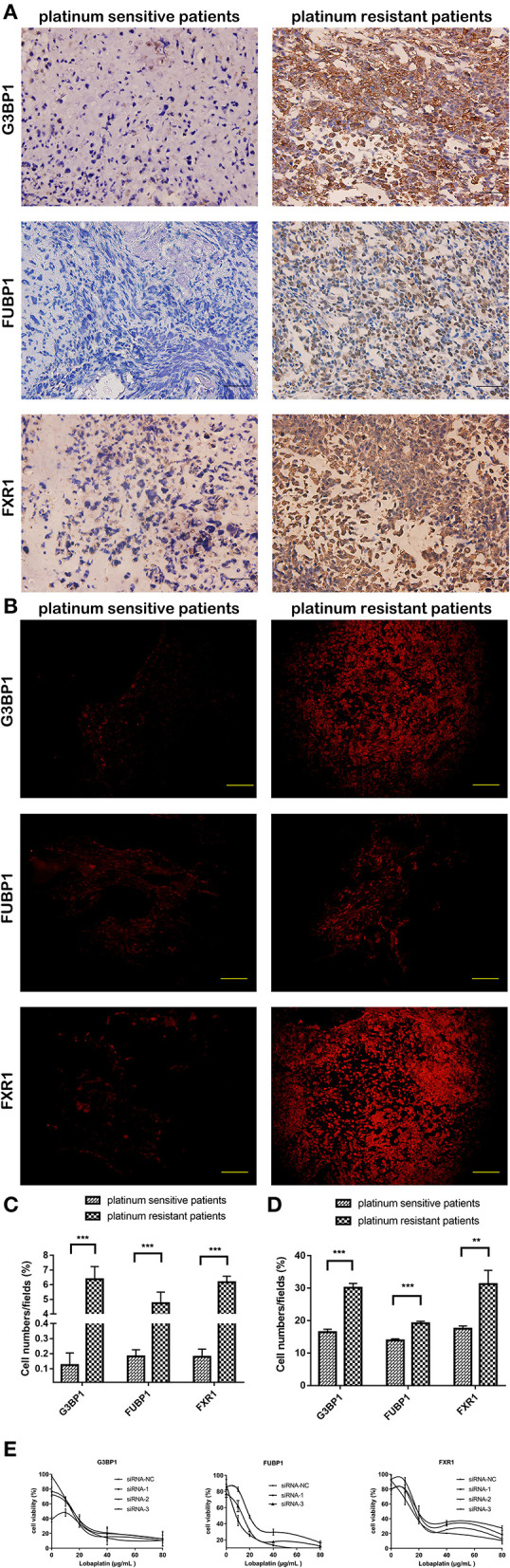
Expression of three candidate proteins show difference between clinical platinum-sensitive and platinum-resistant specimens, while only knockdown of FUBP1 conferred lobaplatin sensitivity in osteosarcoma cells. **(A)** Representative immunohistochemistry staining of clinical specimens; scale bar = 50 μm (magnification, ×400). **(B)** Representative immunofluorescence staining of clinical specimens; scale bar = 100 μm (magnification, ×200). **(C)** Quantification of the expression of the three proteins in immunohistochemistry staining. **(D)** Quantification of the expression of the three proteins in immunofluorescence staining. **(E)** IC50 of lobaplatin in osteosarcoma SaOS-2 cells which were transfected with siRNAs targeting G3BP1, FUBP, and FXR1. Each bar represents the mean ± SEM of three independent experiments. **P < 0.01, ***P < 0.001.

Thirty osteosarcoma specimens, including 15 platinum-based chemotherapy-sensitive and 15 chemotherapy-resistant patients, were detected to assure the PRM results. The clinical data of the specimens are recorded and listed in Supplementary Table 3. The expression of G3BP1, FUBP1, and hFXR1p was strongly positive in 10, 9, and 10 platinum-based chemotherapy-resistant specimens, while in platinum-based chemotherapy-sensitive specimens, these proteins were negative, weakly positive, and at most moderately positive, respectively. The immunofluorescence staining results were in accordance with the immunohistochemistry staining results (Figures 7A–D).

### FUBP1 Knockdown Conferred Lobaplatin Sensitivity of Osteosarcoma SaOS-2 Cells

The three proteins' genes were separately silenced by siRNAs, and the viability of osteosarcoma SaOS-2 cells that were then treated with lobaplatin as described before was measured by CCK8 assay. The results showed that the IC50 values for lobaplatin were strikingly decreased in the FUBP1-silenced cells (RNAi#1, *p* < 0.05), while the IC50 values were not significantly changed in the G3BP1 and FXR1-silenced cells (Figure 7E).

## Discussion

Previous studies have indicated that exogenous and autocrine IL-6 confers chemotherapeutic resistance against platinum-based drugs (6, 15, 16), which is similar to the inference from our CCK-8 and flow cytometry assay in SaOS-2 and SOSP-9607 osteosarcoma cells. However, no comprehensive mechanisms have been explored from the protein perspective. Undoubtedly, proteins are ultimate performers of physiological function as well as a direct reflection of life activity, which is dynamic and flexible. Therefore, investigations into proteins and PPIs are conducive to understanding the whole story. In this study, we demonstrated that IL-6 intervention could decrease the sensitivity of SaOS-2 and SOSP-9607 osteosarcoma cells to lobaplatin. Additionally, a large-scale proteomic analysis among tumor cells in the CG, LG, and rhILG was performed using TMT labeling LC-MS/MS, which could overcome the shortcomings of traditional mass MS after 2D-PAGE and obtain more macromolecules and proteins.

This is the first study using TMT label-based LC-MS/MS technology on IL-6 intervention before osteosarcoma cells were treated with lobaplatin. In the present work, 1,251 proteins with significant differential expression were found between the LG and CG, and 62 proteins with significant differential expression were identified when comparing the LG and rhILG. Namely, there are a host of differentially expressed proteins when cells are treated with lobaplatin compared to control tumor cells, while no more than 100 proteins were differentially expressed between cells treated with lobaplatin and those pretreated with IL-6 before lobaplatin treatment. These results suggest that better homogeneity could be found in cell models than in tissues, which might show complicated heterogeneity; thus, we were able to acquire more accurate results instead of looking for a needle in a haystack.

GO and KEGG enrichment analyses suggested that IL-6 intervention before osteosarcoma cell treatment with lobaplatin significantly regulated Rho GTPase binding proteins and the tubulin complex as well as the estrogen signaling pathway, apoptosis pathway, and IL-17 signaling pathway, which is partly in accordance with our PPI analysis and the flow cytometry and Hoechst staining results. Regarding the estrogen signaling pathway, another study suggested that estrogen can restore tamoxifen sensitivity in breast cancer cells by inducing apoptosis in tamoxifen-resistant cells (17), which explained the enrichment of the estrogen and apoptosis pathways by KEGG analysis. Hence, we speculate that the estrogen and apoptosis pathways may indicate potential therapeutic targets for lobaplatin-resistant osteosarcomas. IL-6 presumably decreased the chemosensitivity of osteosarcoma cells to lobaplatin through estrogen, apoptosis or IL-17 signaling, and proteins involved in the above pathways, such as proto-oncogene, inositol 1,4,5-trisphosphate receptor type 1, keratin, type I cytoskeletal 17, and cytochrome c, may represent other potential targets for lobaplatin-resistant osteosarcoma. This speculation needs to be verified by further experiments in a following study. Interestingly, we also found that two proteins are present in both the estrogen and apoptosis signaling pathways. This is the first report suggesting changes in estrogen as a possible pathway for IL-6-induced resistance to lobaplatin in an osteosarcoma cell model.

The candidate protein G3BP1 showed the highest connectivity with other differentially expressed proteins between the LG and rhILG using both IntAct and STRING. Ras GTPase-activating protein-binding protein 1 (G3BP1) is known to play an essential role in innate immunity as well as ras protein signal transduction. Moreover, it also promotes tumor progression and metastases (18–20). Additionally, it was reported that blocking the functions of ras GTPase-activating protein SH3 domain-binding protein (G3BP) can markedly suppress colon carcinoma HCT116 cell growth, and the downregulation of G3BP could enhance cisplatin-induced apoptosis (21). Although this protein was not enriched to the apoptosis pathway, it is related with cell growth and death to some extent. In our study, the expression of G3BP1 decreased in the LG compared with the CG and increased in rhIL-6-pretreated osteosarcoma cells compared with tumor cells treated with lobaplatin alone. The above finding suggests that changes in this protein are involved in the reduced susceptibility of osteosarcoma to lobaplatin.

Another protein, FUBP1, has been described as an oncoprotein in solid tumor entities as well as many other cancers, promoting tumorigenesis, proliferation, and metastasis of malignant cells (22–26). The silencing of FUBP1 could advance chemosensitivity to adriamycin in gastric cancer (27), and the expression of this protein in B-cell non-Hodgkin lymphoma is associated with cell adhesion-mediated drug resistance (28). Additionally, the UniProt database shows that it can transcriptionally activate the expression of cyclin-D2. In this study, PRM analysis verified that the expression of FUBP1 in rhILG cells was elevated compared with that in LG cells. Far upstream element-binding protein 1 expression was also related to the phenotype of reduced susceptibility to lobaplatin, indicating that it may contribute to reduced sensitivity induced by the cytokine IL-6.

Fragile X mental retardation syndrome-related protein 1 is a highly conserved RNA-binding protein known for its role in muscle development, inflammation, tumorigenesis, and metastatic behavior (29–32) that was enriched for the tubulin complex in the cellular compartment category (GO: 0045298) in our study. A genome-wide RNAi screen identified a host of potential drug resistance genes, including FXR1, by Attila (33). Moreover, Vaquero found that activation of FXR1 enhances chemoresistance of liver tumors against genotoxic compounds (34). Nevertheless, the role of hFXR1p in the drug resistance of osteosarcoma cells to lobaplatin, especially in the presence of IL-6, is unclear. Our proteomics-based study as well as PRM validation demonstrated that hFXR1p is a prominently increased protein in the rhILG compared with the LG, indicating that hFXR1p may be involved in lobaplatin resistance induced by exogenous IL-6 in osteosarcoma cells. The identification of the novel proteins above could lead to the discovery of new drug targets.

Immunohistochemical and immunofluorescence staining were further performed to verify the above three proteins in osteosarcoma patients who underwent platinum-based chemotherapy. Nearly 2/3 specimens from the chemotherapy-resistant group showed extremely strong positive expression of the three proteins, while negative to moderately positive expression was exhibited in the chemotherapy-sensitive group. However, the differential expression of the three proteins in clinical specimens only suggests a correlation between the proteins and the clinical phenomenon. Actually, they may contribute to drug resistance or they are the results of drug resistance.

siRNAs were synthesized to separately silence the three genes, *G3BP1, FUBP1*, and *FXR1*, and the sensitivity of osteosarcoma cells to lobaplatin were detected. Our study demonstrated that only FUBP1-silenced cells showed a decreased IC50 for lobaplatin. Combined with the proteomics and bioinformatics results, as well as the immunohistochemistry staining in clinical specimens, we can conclude that FUBP1 is a special protein that confers lobaplatin resistance in osteosarcoma cells. These findings collectively provide further proof of the role of FUBP1 in the drug susceptibility of osteosarcoma as well as the potential therapeutic value for increasing the sensitivity to lobaplatin in patients with osteosarcoma.

In conclusion, we noted that exogenous IL-6 intervention before lobaplatin treatment resulted in reduced sensitivity of osteosarcoma cells. TMT labeling LC-MS/MS and the subsequent PRM analysis reported herein point to three proteins, including G3BP1, FUBP1, and hFXR1p, as predictive markers to predict lobaplatin responsiveness and clinical outcomes. Although the immunohistochemistry verification was limited to a relatively smaller cohort due to the rarity of osteosarcoma patients who could meet the inclusion criteria, the above proteins present differential expression in platinum-resistant and platinum-sensitive specimens. Separate knockdown of the three genes verified that FUBP1 holds exciting potential as a new target protein to sensitize osteosarcoma cells to lobaplatin treatment. Further studies including larger patient cohorts are needed to clinically evaluate this protein.

## Data Availability Statement

The mass spectrometry proteomics data have been deposited to the ProteomeXchange Consortium via the PRIDE (35) partner repository with the dataset identifier ProteomeXchange: PXD021564.

## Ethics Statement

The studies involving human participants were reviewed and approved by the Institutional Review Board of Tangdu Hospital, Fourth Military Medical University (permission number: TDLL-201903-30). Written informed consent to participate in this study was provided by the participants' legal guardian/next of kin.

## Author Contributions

QM conceived and designed the study. HW and BL analyzed the data and prepared the manuscript. YWu performed *in vitro* experiments in cell lines. KY collected clinical specimens and information. YWe and YL participated in IHC and IF staining. QM and PF provided financial support. All authors contributed to the article and approved the submitted version.

## Conflict of Interest

The authors declare that the research was conducted in the absence of any commercial or financial relationships that could be construed as a potential conflict of interest.
