# Supplementary material for: Protein and Signaling Pathway Responses to rhIL-6 Intervention Before Lobaplatin Treatment in Osteosarcoma Cells
Source: Front Oncol. 2021 Mar 9;11:602712. doi: 10.3389/fonc.2021.602712 (PMC8006349; doi:10.3389/fonc.2021.602712)
Supplement: Supplementary file 1 [file Data_Sheet_1.PDF]

Supplementary Material

Supplementary Figures and Tables

1. Supplementary Figures

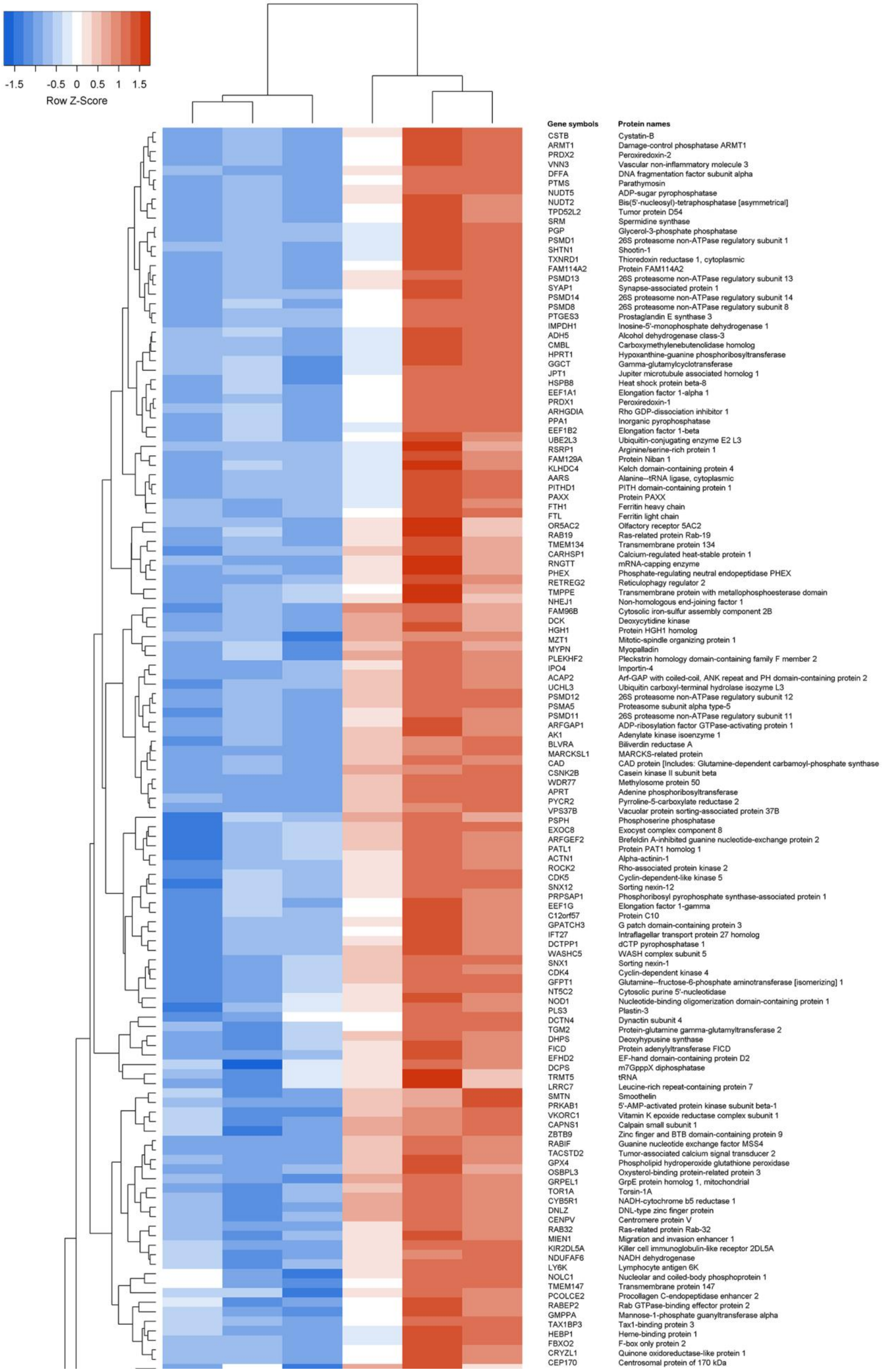
-1.5
-0.5
0
0.5
1
1.5
Row Z-Score
Gene symbols
Protein names
CSTB Cystatin-B
ARMT1 Damage-control phosphatase ARMT1
PRDX2 Peroxiredoxin-2
VNN3 Vascular non-inflammatory molecule 3
DFFA DNA fragmentation factor subunit alpha
PTMS Parathymosin
NUDT5 ADP-sugar pyrophosphatase
NUDT2 Bis(5'-nucleosyl)-tetraphosphatase [asymmetrical]
TPD52L2 Tumor protein D54
SRM Spermidine synthase
PGP Glycerol-3-phosphate phosphatase
PSMD1 26S proteasome non-ATPase regulatory subunit 1
SHTN1 Shootin-1
TXNRD1 Thioredoxin reductase 1, cytoplasmic
FAM114A2 Protein FAM114A2
PSMD13 26S proteasome non-ATPase regulatory subunit 13
SYAP1 Synapse-associated protein 1
PSMD14 26S proteasome non-ATPase regulatory subunit 14
PSMD8 26S proteasome non-ATPase regulatory subunit 8
PTGES3 Prostaglandin E synthase 3
IMPDH1 Inosine-5'-monophosphate dehydrogenase 1
ADH5 Alcohol dehydrogenase class-3
CMBL Carboxymethylenebutenolidase homolog
HPRT1 Hypoxanthine-guanine phosphoribosyltransferase
GGCT Gamma-glutamylcyclotransferase
JPT1 Jupiter microtubule associated homolog 1
HSPB8 Heat shock protein beta-8
EEF1A1 Elongation factor 1-alpha 1
PRDX1 Peroxiredoxin-1
ARHGDIA Rho GDP-dissociation inhibitor 1
PPA1 Inorganic pyrophosphatase
EEF1B2 Elongation factor 1-beta
UBE2L3 Ubiquitin-conjugating enzyme E2 L3
RSRP1 Arginine/serine-rich protein 1
FAM129A Protein Niban 1
KLHDC4 Kelch domain-containing protein 4
AARS Alanine--tRNA ligase, cytoplasmic
PITHD1 PITH domain-containing protein 1
PAXX Protein PAXX
FTH1 Ferritin heavy chain
FTL Ferritin light chain
OR5AC2 Olfactory receptor 5AC2
RAB19 Ras-related protein Rab-19
TMEM134 Transmembrane protein 134
CARHSP1 Calcium-regulated heat-stable protein 1
RNGTT mRNA-capping enzyme
PHEX Phosphate-regulating neutral endopeptidase PHEX
RETREG2 Reticulophagy regulator 2
TMPPE Transmembrane protein with metallophosphoesterase domain
NHEJ1 Non-homologous end-joining factor 1
FAM96B Cytosolic iron-sulfur assembly component 2B
DCK Deoxycytidine kinase
HGH1 Protein HGH1 homolog
MZT1 Mitotic-spindle organizing protein 1
MYPN Myopalladin
PLEKHF2 Pleckstrin homology domain-containing family F member 2
IPO4 Importin-4
ACAP2 Arf-GAP with coiled-coil, ANK repeat and PH domain-containing protein 2
UCHL3 Ubiquitin carboxyl-terminal hydrolase isozyme L3
PSMD12 26S proteasome non-ATPase regulatory subunit 12
PSMA5 Proteasome subunit alpha type-5
PSMD11 26S proteasome non-ATPase regulatory subunit 11
ARFGAP1 ADP-ribosylation factor GTPase-activating protein 1
AK1 Adenylate kinase isoenzyme 1
BLVRA Biliverdin reductase A
MARCKSL1 MARCKS-related protein
CAD CAD protein [Includes: Glutamine-dependent carbamoyl-phosphate synthase
CSNK2B Casein kinase II subunit beta
WDR77 Methylosome protein 50
APRT Adenine phosphoribosyltransferase
PYCR2 Pyrroline-5-carboxylate reductase 2
VPS37B Vacuolar protein sorting-associated protein 37B
PSPH Phosphoserine phosphatase
EXOC8 Exocyst complex component 8
ARFGEF2 Brefeldin A-inhibited guanine nucleotide-exchange protein 2
PATL1 Protein PAT1 homolog 1
ACTN1 Alpha-actinin-1
ROCK2 Rho-associated protein kinase 2
CDK5 Cyclin-dependent-like kinase 5
SNX12 Sorting nexin-12
PRPSAP1 Phosphoribosyl pyrophosphate synthase-associated protein 1
EEF1G Elongation factor 1-gamma
C12orf57 Protein C10
GPATCH3 G patch domain-containing protein 3
IFT27 Intraflagellar transport protein 27 homolog
DCTPP1 dCTP pyrophosphatase 1
WASHC5 WASH complex subunit 5
SNX1 Sorting nexin-1
CDK4 Cyclin-dependent kinase 4
GFPT1 Glutamine--fructose-6-phosphate aminotransferase [isomerizing] 1
NT5C2 Cytosolic purine 5'-nucleotidase
NOD1 Nucleotide-binding oligomerization domain-containing protein 1
PLS3 Plastin-3
DCTN4 Dynactin subunit 4
TGM2 Protein-glutamine gamma-glutamyltransferase 2
DHPS Deoxyhypusine synthase
FICD Protein adenylyltransferase FICD
EFHD2 EF-hand domain-containing protein D2
DCPS m7GpppX diphosphatase
TRMT5 tRNA
LRRC7 Leucine-rich repeat-containing protein 7
SMTN Smoothelin
PRKAB1 5'-AMP-activated protein kinase subunit beta-1
VKORC1 Vitamin K epoxide reductase complex subunit 1
CAPNS1 Calpain small subunit 1
ZBTB9 Zinc finger and BTB domain-containing protein 9
RABIF Guanine nucleotide exchange factor MSS4
TACSTD2 Tumor-associated calcium signal transducer 2
GPX4 Phospholipid hydroperoxide glutathione peroxidase
OSBPL3 Oxysterol-binding protein-related protein 3
GRPEL1 GrpE protein homolog 1, mitochondrial
TOR1A Torsin-1A
CYB5R1 NADH-cytochrome b5 reductase 1
DNLZ DNL-type zinc finger protein
CENPV Centromere protein V
RAB32 Ras-related protein Rab-32
MIEN1 Migration and invasion enhancer 1
KIR2DL5A Killer cell immunoglobulin-like receptor 2DL5A
NDUFAF6 NADH dehydrogenase
LY6K Lymphocyte antigen 6K
NOLC1 Nucleolar and coiled-body phosphoprotein 1
TMEM147 Transmembrane protein 147
PCOLCE2 Procollagen C-endopeptidase enhancer 2
RABEP2 Rab GTPase-binding effector protein 2
GMPPA Mannose-1-phosphate guanyltransferase alpha
TAX1BP3 Tax1-binding protein 3
HEBP1 Heme-binding protein 1
FBXO2 F-box only protein 2
CRYZL1 Quinone oxidoreductase-like protein 1
CEP170 Centrosomal protein of 170 kDa

Fig S1. *Cont.*

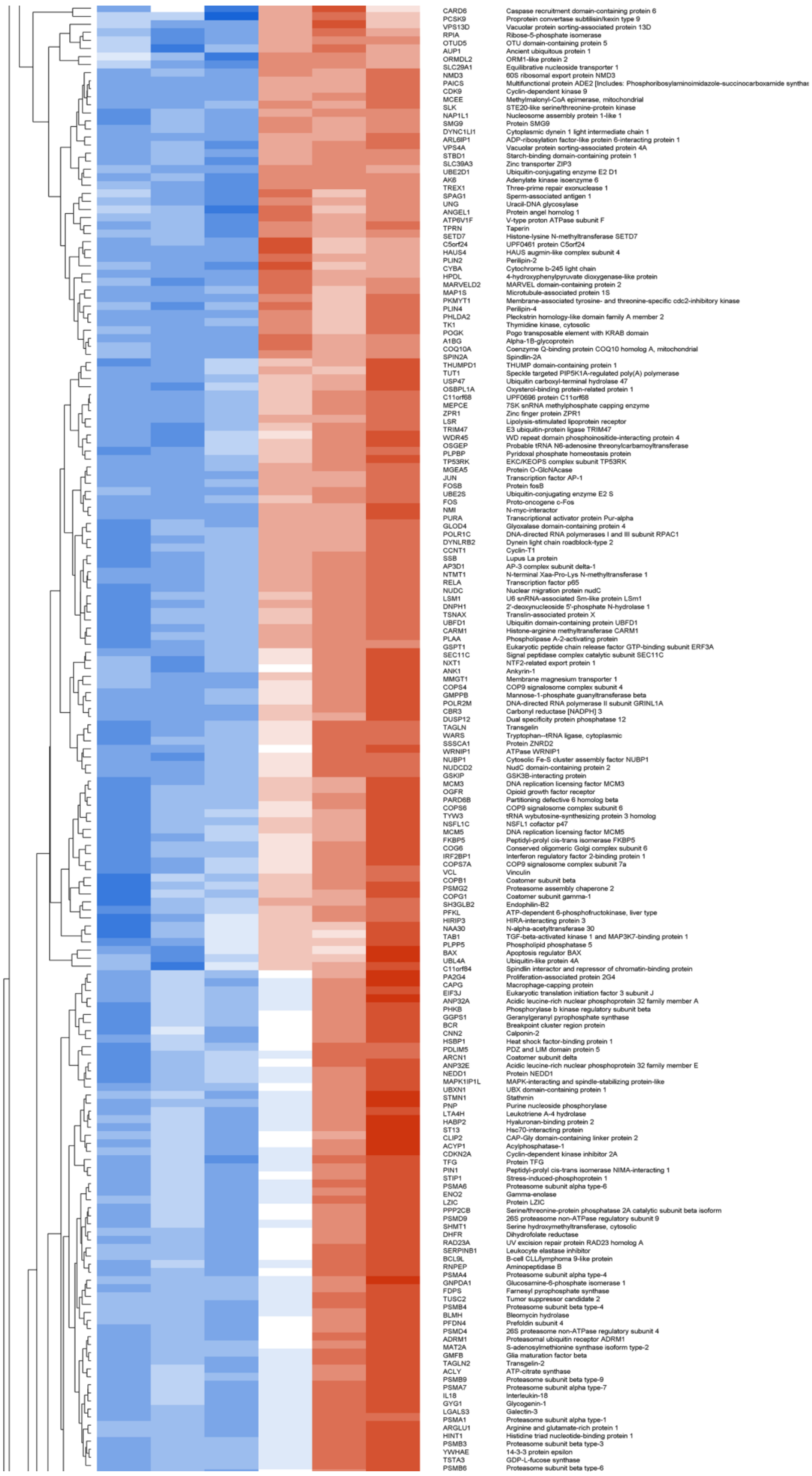

CARD6 Caspase recruitment domain-containing protein 6
PCSK9 Proprotein convertase subtilisin/kexin type 9
VPS13D Vacuolar protein sorting-associated protein 13D
RPIA Ribose-5-phosphate isomerase
OTUD5 OTU domain-containing protein 5
AUP1 Ancient ubiquitous protein 1
ORMDL2 ORM1-like protein 2
SLC29A1 Equilibrative nucleoside transporter 1
NMD3 60S ribosomal export protein NMD3
PAICS Multifunctional protein ADE2 [Includes: Phosphoribosylaminoimidazole-succinocarboxamide synthas
CDK9 Cyclin-dependent kinase 9
MCEE Methylmalonyl-CoA epimerase, mitochondrial
SLK STE20-like serine/threonine-protein kinase
NAP1L1 Nucleosome assembly protein 1-like 1
SMG9 Protein SMG9
DYNC1LI1 Cytoplasmic dynein 1 light intermediate chain 1
ARL6IP1 ADP-ribosylation factor-like protein 6-interacting protein 1
VPS4A Vacuolar protein sorting-associated protein 4A
STBD1 Starch-binding domain-containing protein 1
SLC39A3 Zinc transporter ZIP3
UBE2D1 Ubiquitin-conjugating enzyme E2 D1
AK6 Adenylate kinase isoenzyme 6
TREX1 Three-prime repair exonuclease 1
SPAG1 Sperm-associated antigen 1
UNG Uracil-DNA glycosylase
ANGEL1 Protein angel homolog 1
ATP6V1F V-type proton ATPase subunit F
TPRN Taperin
SETD7 Histone-lysine N-methyltransferase SETD7
C5orf24 UPF0461 protein C5orf24
HAUS4 HAUS augmin-like complex subunit 4
PLIN2 Perilipin-2
CYBA Cytochrome b-245 light chain
HPDL 4-hydroxyphenylpyruvate dioxygenase-like protein
MARVELD2 MARVEL domain-containing protein 2
MAP1S Microtubule-associated protein 1S
PKMYT1 Membrane-associated tyrosine- and threonine-specific cdc2-inhibitory kinase
PLIN4 Perilipin-4
PHLDA2 Pleckstrin homology-like domain family A member 2
TK1 Thymidine kinase, cytosolic
POGK Pogo transposable element with KRAB domain
A1BG Alpha-1B-glycoprotein
COQ10A Coenzyme Q-binding protein COQ10 homolog A, mitochondrial
SPIN2A Spindlin-2A
THUMPD1 THUMP domain-containing protein 1
TUT1 Speckle targeted PIP5K1A-regulated poly(A) polymerase
USP47 Ubiquitin carboxyl-terminal hydrolase 47
OSBPL1A Oxysterol-binding protein-related protein 1
C11orf68 UPF0696 protein C11orf68
MEPCE 7SK snRNA methylphosphate capping enzyme
ZPR1 Zinc finger protein ZPR1
LSR Lipolysis-stimulated lipoprotein receptor
TRIM47 E3 ubiquitin-protein ligase TRIM47
WDR45 WD repeat domain phosphoinositide-interacting protein 4
OSGEP Probable tRNA N6-adenosine threonylcarbamoyltransferase
PLPBP Pyridoxal phosphate homeostasis protein
TP53RK EKC/KEOPS complex subunit TP53RK
MGEA5 Protein O-GlcNAcase
JUN Transcription factor AP-1
FOSB Protein fosB
UBE2S Ubiquitin-conjugating enzyme E2 S
FOS Proto-oncogene c-Fos
NMI N-myc-interactor
PURA Transcriptional activator protein Pur-alpha
GLOD4 Glyoxalase domain-containing protein 4
POLR1C DNA-directed RNA polymerases I and III subunit RPAC1
DYNLRB2 Dynein light chain roadblock-type 2
CCNT1 Cyclin-T1
SSB Lupus La protein
AP3D1 AP-3 complex subunit delta-1
NTMT1 N-terminal Xaa-Pro-Lys N-methyltransferase 1
RELA Transcription factor p65
NUDC Nuclear migration protein nudC
LSM1 U6 snRNA-associated Sm-like protein LSm1
DNPH1 2'-deoxynucleoside 5'-phosphate N-hydrolase 1
TSNAX Translin-associated protein X
UBFD1 Ubiquitin domain-containing protein UBFD1
CARM1 Histone-arginine methyltransferase CARM1
PLAA Phospholipase A-2-activating protein
GSPT1 Eukaryotic peptide chain release factor GTP-binding subunit ERF3A
SEC11C Signal peptidase complex catalytic subunit SEC11C
NXT1 NTF2-related export protein 1
ANK1 Ankyrin-1
MMGT1 Membrane magnesium transporter 1
COPS4 COP9 signalosome complex subunit 4
GMPPB Mannose-1-phosphate guanyltransferase beta
POLR2M DNA-directed RNA polymerase II subunit GRINL1A
CBR3 Carbonyl reductase [NADPH] 3
DUSP12 Dual specificity protein phosphatase 12
TAGLN Transgelin
WARS Tryptophan--tRNA ligase, cytoplasmic
SSSCA1 Protein ZNRD2
WRNIP1 ATPase WRNIP1
NUBP1 Cytosolic Fe-S cluster assembly factor NUBP1
NUDCD2 NudC domain-containing protein 2
GSKIP GSK3B-interacting protein
MCM3 DNA replication licensing factor MCM3
OGFR Opioid growth factor receptor
PARD6B Partitioning defective 6 homolog beta
COPS6 COP9 signalosome complex subunit 6
TYW3 tRNA wybutosine-synthesizing protein 3 homolog
NSFL1C NSFL1 cofactor p47
MCM5 DNA replication licensing factor MCM5
FKBP5 Peptidyl-prolyl cis-trans isomerase FKBP5
COG6 Conserved oligomeric Golgi complex subunit 6
IRF2BP1 Interferon regulatory factor 2-binding protein 1
COPS7A COP9 signalosome complex subunit 7a
VCL Vinculin
COPB1 Coatomer subunit beta
PSMG2 Proteasome assembly chaperone 2
COPG1 Coatomer subunit gamma-1
SH3GLB2 Endophilin-B2
PFKL ATP-dependent 6-phosphofructokinase, liver type
HIRIP3 HIRA-interacting protein 3
NAA30 N-alpha-acetyltransferase 30
TAB1 TGF-beta-activated kinase 1 and MAP3K7-binding protein 1
PLPP5 Phospholipid phosphatase 5
BAX Apoptosis regulator BAX
UBL4A Ubiquitin-like protein 4A
C11orf84 Spindlin interactor and repressor of chromatin-binding protein
PA2G4 Proliferation-associated protein 2G4
CAPG Macrophage-capping protein
EIF3J Eukaryotic translation initiation factor 3 subunit J
ANP32A Acidic leucine-rich nuclear phosphoprotein 32 family member A
PHKB Phosphorylase b kinase regulatory subunit beta
GGPS1 Geranylgeranyl pyrophosphate synthase
BCR Breakpoint cluster region protein
CNN2 Calponin-2
HSBP1 Heat shock factor-binding protein 1
PDLIM5 PDZ and LIM domain protein 5
ARCN1 Coatomer subunit delta
ANP32E Acidic leucine-rich nuclear phosphoprotein 32 family member E
NEDD1 Protein NEDD1
MAPK1IP1L MAPK-interacting and spindle-stabilizing protein-like
UBXN1 UBX domain-containing protein 1
STMN1 Stathmin
PNP Purine nucleoside phosphorylase
LTA4H Leukotriene A-4 hydrolase
HABP2 Hyaluronan-binding protein 2
ST13 Hsc70-interacting protein
CLIP2 CAP-Gly domain-containing linker protein 2
ACYP1 Acylphosphatase-1
CDKN2A Cyclin-dependent kinase inhibitor 2A
TFG Protein TFG
PIN1 Peptidyl-prolyl cis-trans isomerase NIMA-interacting 1
STIP1 Stress-induced-phosphoprotein 1
PSMA6 Proteasome subunit alpha type-6
ENO2 Gamma-enolase
LZIC Protein LZIC
PPP2CB Serine/threonine-protein phosphatase 2A catalytic subunit beta isoform
PSMD9 26S proteasome non-ATPase regulatory subunit 9
SHMT1 Serine hydroxymethyltransferase, cytosolic
DHFR Dihydrofolate reductase
RAD23A UV excision repair protein RAD23 homolog A
SERPINB1 Leukocyte elastase inhibitor
BCL9L B-cell CLL/lymphoma 9-like protein
RNPEP Aminopeptidase B
PSMA4 Proteasome subunit alpha type-4
GNPDA1 Glucosamine-6-phosphate isomerase 1
FDPS Farnesyl pyrophosphate synthase
TUSC2 Tumor suppressor candidate 2
PSMB4 Proteasome subunit beta type-4
BLMH Bleomycin hydrolase
PFDN4 Prefoldin subunit 4
PSMD4 26S proteasome non-ATPase regulatory subunit 4
ADRM1 Proteasomal ubiquitin receptor ADRM1
MAT2A S-adenosylmethionine synthase isoform type-2
GMFB Glia maturation factor beta
TAGLN2 Transgelin-2
ACLY ATP-citrate synthase
PSMB9 Proteasome subunit beta type-9
PSMA7 Proteasome subunit alpha type-7
IL18 Interleukin-18
GYG1 Glycogenin-1
LGALS3 Galectin-3
PSMA1 Proteasome subunit alpha type-1
ARGLU1 Arginine and glutamate-rich protein 1
HINT1 Histidine triad nucleotide-binding protein 1
PSMB3 Proteasome subunit beta type-3
YWHAE 14-3-3 protein epsilon
TSTA3 GDP-L-fucose synthase
PSMB6 Proteasome subunit beta type-6

Fig S1. *Cont.*

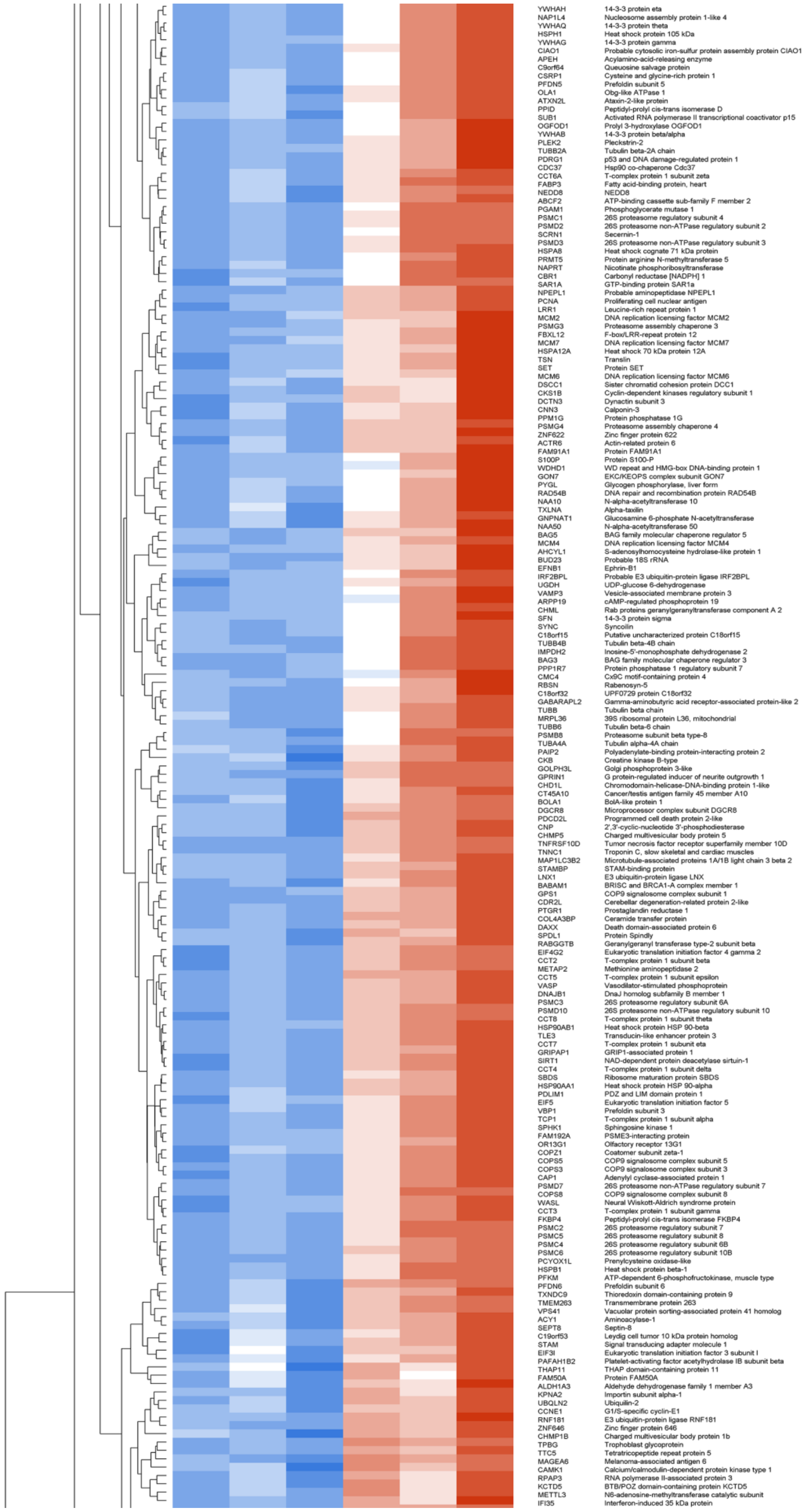
YWHAH 14-3-3 protein eta
NAP1L4 Nucleosome assembly protein 1-like 4
YWHAQ 14-3-3 protein theta
HSPH1 Heat shock protein 105 kDa
YWHAG 14-3-3 protein gamma
CIAO1 Probable cytosolic iron-sulfur protein assembly protein CIAO1
APEH Acylamino-acid-releasing enzyme
C9orf64 Queuosine salvage protein
CSRP1 Cysteine and glycine-rich protein 1
PFDN5 Prefoldin subunit 5
OLA1 Obg-like ATPase 1
ATXN2L Ataxin-2-like protein
PPID Peptidyl-prolyl cis-trans isomerase D
SUB1 Activated RNA polymerase II transcriptional coactivator p15
OGFOD1 Prolyl 3-hydroxylase OGFOD1
YWHAB 14-3-3 protein beta/alpha
PLEK2 Pleckstrin-2
TUBB2A Tubulin beta-2A chain
PDRG1 p53 and DNA damage-regulated protein 1
CDC37 Hsp90 co-chaperone Cdc37
CCT6A T-complex protein 1 subunit zeta
FABP3 Fatty acid-binding protein, heart
NEDD8 NEDD8
ABCF2 ATP-binding cassette sub-family F member 2
PGAM1 Phosphoglycerate mutase 1
PSMC1 26S proteasome regulatory subunit 4
PSMD2 26S proteasome non-ATPase regulatory subunit 2
SCRN1 Secernin-1
PSMD3 26S proteasome non-ATPase regulatory subunit 3
HSPA8 Heat shock cognate 71 kDa protein
PRMT5 Protein arginine N-methyltransferase 5
NAPRT Nicotinate phosphoribosyltransferase
CBR1 Carbonyl reductase [NADPH] 1
SAR1A GTP-binding protein SAR1a
NPEPL1 Probable aminopeptidase NPEPL1
PCNA Proliferating cell nuclear antigen
LRR1 Leucine-rich repeat protein 1
MCM2 DNA replication licensing factor MCM2
PSMG3 Proteasome assembly chaperone 3
FBXL12 F-box/LRR-repeat protein 12
MCM7 DNA replication licensing factor MCM7
HSPA12A Heat shock 70 kDa protein 12A
TSN Translin
SET Protein SET
MCM6 DNA replication licensing factor MCM6
DSCC1 Sister chromatid cohesion protein DCC1
CKS1B Cyclin-dependent kinases regulatory subunit 1
DCTN3 Dynactin subunit 3
CNN3 Calponin-3
PPM1G Protein phosphatase 1G
PSMG4 Proteasome assembly chaperone 4
ZNF622 Zinc finger protein 622
ACTR6 Actin-related protein 6
FAM91A1 Protein FAM91A1
S100P Protein S100-P
WDHD1 WD repeat and HMG-box DNA-binding protein 1
GON7 EKC/KEOPS complex subunit GON7
PYGL Glycogen phosphorylase, liver form
RAD54B DNA repair and recombination protein RAD54B
NAA10 N-alpha-acetyltransferase 10
TXLNA Alpha-taxilin
GNPNAT1 Glucosamine 6-phosphate N-acetyltransferase
NAA50 N-alpha-acetyltransferase 50
BAG5 BAG family molecular chaperone regulator 5
MCM4 DNA replication licensing factor MCM4
AHCYL1 S-adenosylhomocysteine hydrolase-like protein 1
BUD23 Probable 18S rRNA
EFNB1 Ephrin-B1
IRF2BPL Probable E3 ubiquitin-protein ligase IRF2BPL
UGDH UDP-glucose 6-dehydrogenase
VAMP3 Vesicle-associated membrane protein 3
ARPP19 cAMP-regulated phosphoprotein 19
CHML Rab proteins geranylgeranyltransferase component A 2
SFN 14-3-3 protein sigma
SYNC Syncoilin
C18orf15 Putative uncharacterized protein C18orf15
TUBB4B Tubulin beta-4B chain
IMPDH2 Inosine-5'-monophosphate dehydrogenase 2
BAG3 BAG family molecular chaperone regulator 3
PPP1R7 Protein phosphatase 1 regulatory subunit 7
CMC4 Cx9C motif-containing protein 4
RBSN Rabenosyn-5
C18orf32 UPF0729 protein C18orf32
GABARAPL2 Gamma-aminobutyric acid receptor-associated protein-like 2
TUBB Tubulin beta chain
MRPL36 39S ribosomal protein L36, mitochondrial
TUBB6 Tubulin beta-6 chain
PSMB8 Proteasome subunit beta type-8
TUBA4A Tubulin alpha-4A chain
PAIP2 Polyadenylate-binding protein-interacting protein 2
CKB Creatine kinase B-type
GOLPH3L Golgi phosphoprotein 3-like
GPRIN1 G protein-regulated inducer of neurite outgrowth 1
CHD1L Chromodomain-helicase-DNA-binding protein 1-like
CT45A10 Cancer/testis antigen family 45 member A10
BOLA1 BolA-like protein 1
DGCR8 Microprocessor complex subunit DGCR8
PDCD2L Programmed cell death protein 2-like
CNP 2',3'-cyclic-nucleotide 3'-phosphodiesterase
CHMP5 Charged multivesicular body protein 5
TNFRSF10D Tumor necrosis factor receptor superfamily member 10D
TNNC1 Troponin C, slow skeletal and cardiac muscles
MAP1LC3B2 Microtubule-associated proteins 1A/1B light chain 3 beta 2
STAMBP STAM-binding protein
LNX1 E3 ubiquitin-protein ligase LNX
BABAM1 BRISC and BRCA1-A complex member 1
GPS1 COP9 signalosome complex subunit 1
CDR2L Cerebellar degeneration-related protein 2-like
PTGR1 Prostaglandin reductase 1
COL4A3BP Ceramide transfer protein
DAXX Death domain-associated protein 6
SPDL1 Protein Spindly
RABGGTB Geranylgeranyl transferase type-2 subunit beta
EIF4G2 Eukaryotic translation initiation factor 4 gamma 2
CCT2 T-complex protein 1 subunit beta
METAP2 Methionine aminopeptidase 2
CCT5 T-complex protein 1 subunit epsilon
VASP Vasodilator-stimulated phosphoprotein
DNAJB1 DnaJ homolog subfamily B member 1
PSMC3 26S proteasome regulatory subunit 6A
PSMD10 26S proteasome non-ATPase regulatory subunit 10
CCT8 T-complex protein 1 subunit theta
HSP90AB1 Heat shock protein HSP 90-beta
TLE3 Transducin-like enhancer protein 3
CCT7 T-complex protein 1 subunit eta
GRIPAP1 GRIP1-associated protein 1
SIRT1 NAD-dependent protein deacetylase sirtuin-1
CCT4 T-complex protein 1 subunit delta
SBDS Ribosome maturation protein SBDS
HSP90AA1 Heat shock protein HSP 90-alpha
PDLIM1 PDZ and LIM domain protein 1
EIF5 Eukaryotic translation initiation factor 5
VBP1 Prefoldin subunit 3
TCP1 T-complex protein 1 subunit alpha
SPHK1 Sphingosine kinase 1
FAM192A PSME3-interacting protein
OR13G1 Olfactory receptor 13G1
COPZ1 Coatomer subunit zeta-1
COPS5 COP9 signalosome complex subunit 5
COPS3 COP9 signalosome complex subunit 3
CAP1 Adenylyl cyclase-associated protein 1
PSMD7 26S proteasome non-ATPase regulatory subunit 7
COPS8 COP9 signalosome complex subunit 8
WASL Neural Wiskott-Aldrich syndrome protein
CCT3 T-complex protein 1 subunit gamma
FKBP4 Peptidyl-prolyl cis-trans isomerase FKBP4
PSMC2 26S proteasome regulatory subunit 7
PSMC5 26S proteasome regulatory subunit 8
PSMC4 26S proteasome regulatory subunit 6B
PSMC6 26S proteasome regulatory subunit 10B
PCYOX1L Prenylcysteine oxidase-like
HSPB1 Heat shock protein beta-1
PFKM ATP-dependent 6-phosphofructokinase, muscle type
PFDN6 Prefoldin subunit 6
TXNDC9 Thioredoxin domain-containing protein 9
TMEM263 Transmembrane protein 263
VPS41 Vacuolar protein sorting-associated protein 41 homolog
ACY1 Aminoacylase-1
SEPT8 Septin-8
C19orf53 Leydig cell tumor 10 kDa protein homolog
STAM Signal transducing adapter molecule 1
EIF3I Eukaryotic translation initiation factor 3 subunit I
PAFAH1B2 Platelet-activating factor acetylhydrolase IB subunit beta
THAP11 THAP domain-containing protein 11
FAM50A Protein FAM50A
ALDH1A3 Aldehyde dehydrogenase family 1 member A3
KPNA2 Importin subunit alpha-1
UBQLN2 Ubiquilin-2
CCNE1 G1/S-specific cyclin-E1
RNF181 E3 ubiquitin-protein ligase RNF181
ZNF646 Zinc finger protein 646
CHMP1B Charged multivesicular body protein 1b
TPBG Trophoblast glycoprotein
TTC5 Tetratricopeptide repeat protein 5
MAGEA6 Melanoma-associated antigen 6
CAMK1 Calcium/calmodulin-dependent protein kinase type 1
RPAP3 RNA polymerase II-associated protein 3
KCTD5 BTB/POZ domain-containing protein KCTD5
METTL3 N6-adenosine-methyltransferase catalytic subunit
IFI35 Interferon-induced 35 kDa protein

Fig S1. *Cont.*

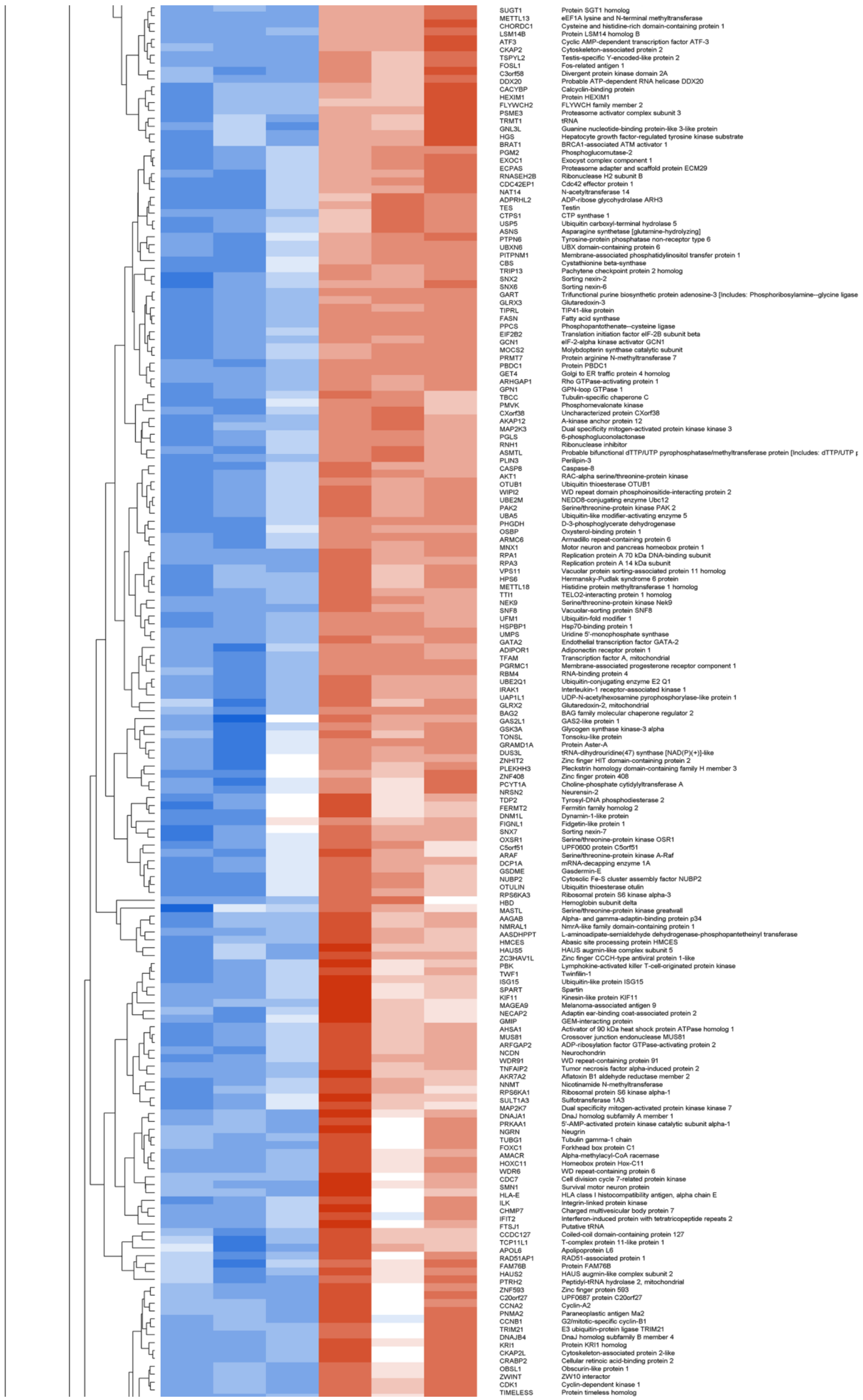

SUGT1 Protein SGT1 homolog
METTL13 eEF1A lysine and N-terminal methyltransferase
CHORDC1 Cysteine and histidine-rich domain-containing protein 1
LSM14B Protein LSM14 homolog B
ATF3 Cyclic AMP-dependent transcription factor ATF-3
CKAP2 Cytoskeleton-associated protein 2
TSPYL2 Testis-specific Y-encoded-like protein 2
FOSL1 Fos-related antigen 1
C3orf58 Divergent protein kinase domain 2A
DDX20 Probable ATP-dependent RNA helicase DDX20
CACYBP Calcyclin-binding protein
HEXIM1 Protein HEXIM1
FLYWCH2 FLYWCH family member 2
PSME3 Proteasome activator complex subunit 3
TRMT1 tRNA
GNL3L Guanine nucleotide-binding protein-like 3-like protein
HGS Hepatocyte growth factor-regulated tyrosine kinase substrate
BRAT1 BRCA1-associated ATM activator 1
PGM2 Phosphoglucomutase-2
EXOC1 Exocyst complex component 1
ECPAS Proteasome adapter and scaffold protein ECM29
RNASEH2B Ribonuclease H2 subunit B
CDC42EP1 Cdc42 effector protein 1
NAT14 N-acetyltransferase 14
ADPRHL2 ADP-ribose glycohydrolase ARH3
TES Testin
CTPS1 CTP synthase 1
USP5 Ubiquitin carboxyl-terminal hydrolase 5
ASNS Asparagine synthetase [glutamine-hydrolyzing]
PTPN6 Tyrosine-protein phosphatase non-receptor type 6
UBXN6 UBX domain-containing protein 6
PITPNM1 Membrane-associated phosphatidylinositol transfer protein 1
CBS Cystathionine beta-synthase
TRIP13 Pachytene checkpoint protein 2 homolog
SNX2 Sorting nexin-2
SNX6 Sorting nexin-6
GART Trifunctional purine biosynthetic protein adenosine-3 [Includes: Phosphoribosylamine--glycine ligase
GLRX3 Glutaredoxin-3
TIPRL TIP41-like protein
FASN Fatty acid synthase
PPCS Phosphopantothenate--cysteine ligase
EIF2B2 Translation initiation factor eIF-2B subunit beta
GCN1 eIF-2-alpha kinase activator GCN1
MOCS2 Molybdopterin synthase catalytic subunit
PRMT7 Protein arginine N-methyltransferase 7
PBDC1 Protein PBDC1
GET4 Golgi to ER traffic protein 4 homolog
ARHGAP1 Rho GTPase-activating protein 1
GPN1 GPN-loop GTPase 1
TBCC Tubulin-specific chaperone C
PMVK Phosphomevalonate kinase
CXorf38 Uncharacterized protein CXorf38
AKAP12 A-kinase anchor protein 12
MAP2K3 Dual specificity mitogen-activated protein kinase kinase 3
PGLS 6-phosphogluconolactonase
RNH1 Ribonuclease inhibitor
ASMTL Probable bifunctional dTTP/UTP pyrophosphatase/methyltransferase protein [Includes: dTTP/UTP p
PLIN3 Perilipin-3
CASP8 Caspase-8
AKT1 RAC-alpha serine/threonine-protein kinase
OTUB1 Ubiquitin thioesterase OTUB1
WIPI2 WD repeat domain phosphoinositide-interacting protein 2
UBE2M NEDD8-conjugating enzyme Ubc12
PAK2 Serine/threonine-protein kinase PAK 2
UBA5 Ubiquitin-like modifier-activating enzyme 5
PHGDH D-3-phosphoglycerate dehydrogenase
OSBP Oxysterol-binding protein 1
ARMC6 Armadillo repeat-containing protein 6
MNX1 Motor neuron and pancreas homeobox protein 1
RPA1 Replication protein A 70 kDa DNA-binding subunit
RPA3 Replication protein A 14 kDa subunit
VPS11 Vacuolar protein sorting-associated protein 11 homolog
HPS6 Hermansky-Pudlak syndrome 6 protein
METTL18 Histidine protein methyltransferase 1 homolog
TTI1 TELO2-interacting protein 1 homolog
NEK9 Serine/threonine-protein kinase Nek9
SNF8 Vacuolar-sorting protein SNF8
UFM1 Ubiquitin-fold modifier 1
HSPBP1 Hsp70-binding protein 1
UMPS Uridine 5'-monophosphate synthase
GATA2 Endothelial transcription factor GATA-2
ADIPOR1 Adiponectin receptor protein 1
TFAM Transcription factor A, mitochondrial
PGRMC1 Membrane-associated progesterone receptor component 1
RBM4 RNA-binding protein 4
UBE2Q1 Ubiquitin-conjugating enzyme E2 Q1
IRAK1 Interleukin-1 receptor-associated kinase 1
UAP1L1 UDP-N-acetylhexosamine pyrophosphorylase-like protein 1
GLRX2 Glutaredoxin-2, mitochondrial
BAG2 BAG family molecular chaperone regulator 2
GAS2L1 GAS2-like protein 1
GSK3A Glycogen synthase kinase-3 alpha
TONSL Tonsoku-like protein
GRAMD1A Protein Aster-A
DUS3L tRNA-dihydrouridine(47) synthase [NAD(P)(+)]-like
ZNHIT2 Zinc finger HIT domain-containing protein 2
PLEKHH3 Pleckstrin homology domain-containing family H member 3
ZNF408 Zinc finger protein 408
PCYT1A Choline-phosphate cytidylyltransferase A
NRSN2 Neurensin-2
TDP2 Tyrosyl-DNA phosphodiesterase 2
FERMT2 Fermitin family homolog 2
DNM1L Dynamin-1-like protein
FIGNL1 Fidgetin-like protein 1
SNX7 Sorting nexin-7
OXSR1 Serine/threonine-protein kinase OSR1
C5orf51 UPF0600 protein C5orf51
ARAF Serine/threonine-protein kinase A-Raf
DCP1A mRNA-decapping enzyme 1A
GSDME Gasdermin-E
NUBP2 Cytosolic Fe-S cluster assembly factor NUBP2
OTULIN Ubiquitin thioesterase otulin
RPS6KA3 Ribosomal protein S6 kinase alpha-3
HBD Hemoglobin subunit delta
MASTL Serine/threonine-protein kinase greatwall
AAGAB Alpha- and gamma-adaptin-binding protein p34
NMRAL1 NmrA-like family domain-containing protein 1
AASDHPPT L-aminoadipate-semialdehyde dehydrogenase-phosphopantetheinyl transferase
HMCES Abasic site processing protein HMCES
HAUS5 HAUS augmin-like complex subunit 5
ZC3HAV1L Zinc finger CCCH-type antiviral protein 1-like
PBK Lymphokine-activated killer T-cell-originated protein kinase
TWF1 Twinfilin-1
ISG15 Ubiquitin-like protein ISG15
SPART Spartin
KIF11 Kinesin-like protein KIF11
MAGEA9 Melanoma-associated antigen 9
NECAP2 Adaptin ear-binding coat-associated protein 2
GMIP GEM-interacting protein
AHSA1 Activator of 90 kDa heat shock protein ATPase homolog 1
MUS81 Crossover junction endonuclease MUS81
ARFGAP2 ADP-ribosylation factor GTPase-activating protein 2
NCDN Neurochondrin
WDR91 WD repeat-containing protein 91
TNFAIP2 Tumor necrosis factor alpha-induced protein 2
AKR7A2 Aflatoxin B1 aldehyde reductase member 2
NNMT Nicotinamide N-methyltransferase
RPS6KA1 Ribosomal protein S6 kinase alpha-1
SULT1A3 Sulfotransferase 1A3
MAP2K7 Dual specificity mitogen-activated protein kinase kinase 7
DNAJA1 DnaJ homolog subfamily A member 1
PRKAA1 5'-AMP-activated protein kinase catalytic subunit alpha-1
NGRN Neugrin
TUBG1 Tubulin gamma-1 chain
FOXC1 Forkhead box protein C1
AMACR Alpha-methylacyl-CoA racemase
HOXC11 Homeobox protein Hox-C11
WDR6 WD repeat-containing protein 6
CDC7 Cell division cycle 7-related protein kinase
SMN1 Survival motor neuron protein
HLA-E HLA class I histocompatibility antigen, alpha chain E
ILK Integrin-linked protein kinase
CHMP7 Charged multivesicular body protein 7
IFIT2 Interferon-induced protein with tetratricopeptide repeats 2
FTSJ1 Putative tRNA
CCDC127 Coiled-coil domain-containing protein 127
TCP11L1 T-complex protein 11-like protein 1
APOL6 Apolipoprotein L6
RAD51AP1 RAD51-associated protein 1
FAM76B Protein FAM76B
HAUS2 HAUS augmin-like complex subunit 2
PTRH2 Peptidyl-tRNA hydrolase 2, mitochondrial
ZNF593 Zinc finger protein 593
C20orf27 UPF0687 protein C20orf27
CCNA2 Cyclin-A2
PNMA2 Paraneoplastic antigen Ma2
CCNB1 G2/mitotic-specific cyclin-B1
TRIM21 E3 ubiquitin-protein ligase TRIM21
DNAJB4 DnaJ homolog subfamily B member 4
KRI1 Protein KRI1 homolog
CKAP2L Cytoskeleton-associated protein 2-like
CRABP2 Cellular retinoic acid-binding protein 2
OBSL1 Obscurin-like protein 1
ZWINT ZW10 interactor
CDK1 Cyclin-dependent kinase 1
TIMELESS Protein timeless homolog

Fig S1. *Cont.*

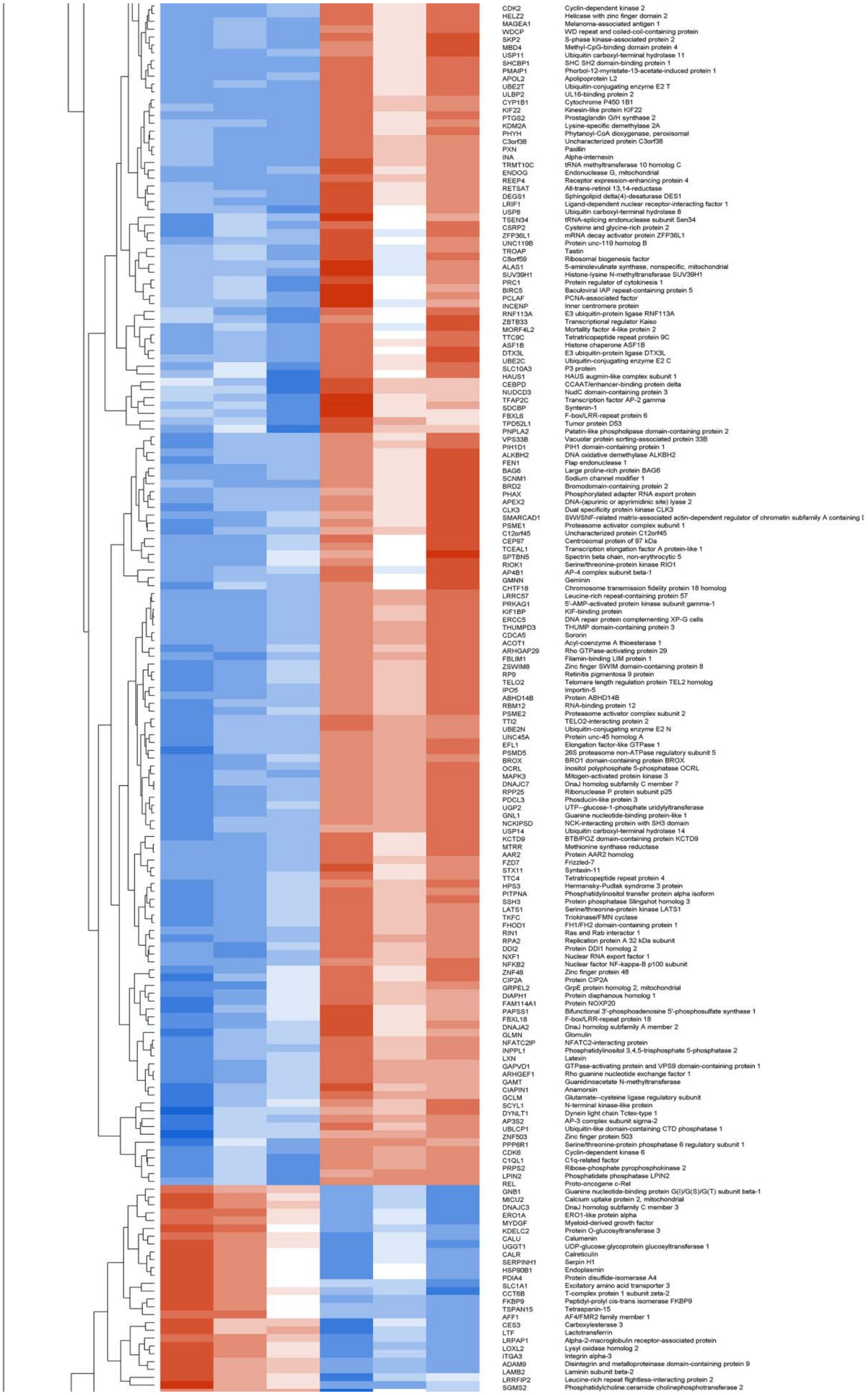

CDK2 Cyclin-dependent kinase 2
HELZ2 Helicase with zinc finger domain 2
MAGEA1 Melanoma-associated antigen 1
WDCP WD repeat and coiled-coil-containing protein
SKP2 S-phase kinase-associated protein 2
MBD4 Methyl-CpG-binding domain protein 4
USP11 Ubiquitin carboxyl-terminal hydrolase 11
SHCBP1 SHC SH2 domain-binding protein 1
PMAIP1 Phorbol-12-myristate-13-acetate-induced protein 1
APOL2 Apolipoprotein L2
UBE2T Ubiquitin-conjugating enzyme E2 T
ULBP2 UL16-binding protein 2
CYP1B1 Cytochrome P450 1B1
KIF22 Kinesin-like protein KIF22
PTGS2 Prostaglandin G/H synthase 2
KDM2A Lysine-specific demethylase 2A
PHYH Phytanoyl-CoA dioxygenase, peroxisomal
C3orf38 Uncharacterized protein C3orf38
PXN Paxillin
INA Alpha-internexin
TRMT10C tRNA methyltransferase 10 homolog C
ENDOG Endonuclease G, mitochondrial
REEP4 Receptor expression-enhancing protein 4
RETSAT All-trans-retinol 13,14-reductase
DEGS1 Sphingolipid delta(4)-desaturase DES1
LRIF1 Ligand-dependent nuclear receptor-interacting factor 1
USP8 Ubiquitin carboxyl-terminal hydrolase 8
TSEN34 tRNA-splicing endonuclease subunit Sen34
CSRP2 Cysteine and glycine-rich protein 2
ZFP36L1 mRNA decay activator protein ZFP36L1
UNC119B Protein unc-119 homolog B
TROAP Tastin
C8orf59 Ribosomal biogenesis factor
ALAS1 5-aminolevulinate synthase, nonspecific, mitochondrial
SUV39H1 Histone-lysine N-methyltransferase SUV39H1
PRC1 Protein regulator of cytokinesis 1
BIRC5 Baculoviral IAP repeat-containing protein 5
PCLAF PCNA-associated factor
INCENP Inner centromere protein
RNF113A E3 ubiquitin-protein ligase RNF113A
ZBTB33 Transcriptional regulator Kaiso
MORF4L2 Mortality factor 4-like protein 2
TTC9C Tetratricopeptide repeat protein 9C
ASF1B Histone chaperone ASF1B
DTX3L E3 ubiquitin-protein ligase DTX3L
UBE2C Ubiquitin-conjugating enzyme E2 C
SLC10A3 P3 protein
HAUS1 HAUS augmin-like complex subunit 1
CEBPD CCAAT/enhancer-binding protein delta
NUDCD3 NudC domain-containing protein 3
TFAP2C Transcription factor AP-2 gamma
SDCBP Syntenin-1
FBXL6 F-box/LRR-repeat protein 6
TPD52L1 Tumor protein D53
PNPLA2 Patatin-like phospholipase domain-containing protein 2
VPS33B Vacuolar protein sorting-associated protein 33B
PIH1D1 PIH1 domain-containing protein 1
ALKBH2 DNA oxidative demethylase ALKBH2
FEN1 Flap endonuclease 1
BAG6 Large proline-rich protein BAG6
SCNM1 Sodium channel modifier 1
BRD2 Bromodomain-containing protein 2
PHAX Phosphorylated adapter RNA export protein
APEX2 DNA-(apurinic or apyrimidinic site) lyase 2
CLK3 Dual specificity protein kinase CLK3
SMARCAD1 SWI/SNF-related matrix-associated actin-dependent regulator of chromatin subfamily A containing
PSME1 Proteasome activator complex subunit 1
C12orf45 Uncharacterized protein C12orf45
CEP97 Centrosomal protein of 97 kDa
TCEAL1 Transcription elongation factor A protein-like 1
SPTBN5 Spectrin beta chain, non-erythrocytic 5
RIOK1 Serine/threonine-protein kinase RIO1
AP4B1 AP-4 complex subunit beta-1
GMNN Geminin
CHTF18 Chromosome transmission fidelity protein 18 homolog
LRRC57 Leucine-rich repeat-containing protein 57
PRKAG1 5'-AMP-activated protein kinase subunit gamma-1
KIF1BP KIF-binding protein
ERCC5 DNA repair protein complementing XP-G cells
THUMPD3 THUMP domain-containing protein 3
CDCA5 Sororin
ACOT1 Acyl-coenzyme A thioesterase 1
ARHGAP29 Rho GTPase-activating protein 29
FBLIM1 Filamin-binding LIM protein 1
ZSWIM8 Zinc finger SWIM domain-containing protein 8
RP9 Retinitis pigmentosa 9 protein
TELO2 Telomere length regulation protein TEL2 homolog
IPO5 Importin-5
ABHD14B Protein ABHD14B
RBM12 RNA-binding protein 12
PSME2 Proteasome activator complex subunit 2
TTI2 TELO2-interacting protein 2
UBE2N Ubiquitin-conjugating enzyme E2 N
UNC45A Protein unc-45 homolog A
EFL1 Elongation factor-like GTPase 1
PSMD5 26S proteasome non-ATPase regulatory subunit 5
BROX BRO1 domain-containing protein BROX
OCRL Inositol polyphosphate 5-phosphatase OCRL
MAPK3 Mitogen-activated protein kinase 3
DNAJC7 DnaJ homolog subfamily C member 7
RPP25 Ribonuclease P protein subunit p25
PDCL3 Phosducin-like protein 3
UGP2 UTP--glucose-1-phosphate uridylyltransferase
GNL1 Guanine nucleotide-binding protein-like 1
NCKIPSD NCK-interacting protein with SH3 domain
USP14 Ubiquitin carboxyl-terminal hydrolase 14
KCTD9 BTB/POZ domain-containing protein KCTD9
MTRR Methionine synthase reductase
AAR2 Protein AAR2 homolog
FZD7 Frizzled-7
STX11 Syntaxin-11
TTC4 Tetratricopeptide repeat protein 4
HPS3 Hermansky-Pudlak syndrome 3 protein
PITPNA Phosphatidylinositol transfer protein alpha isoform
SSH3 Protein phosphatase Slingshot homolog 3
LATS1 Serine/threonine-protein kinase LATS1
TKFC Triokinase/FMN cyclase
FHOD1 FH1/FH2 domain-containing protein 1
RIN1 Ras and Rab interactor 1
RPA2 Replication protein A 32 kDa subunit
DDI2 Protein DDI1 homolog 2
NXF1 Nuclear RNA export factor 1
NFKB2 Nuclear factor NF-kappa-B p100 subunit
ZNF48 Zinc finger protein 48
CIP2A Protein CIP2A
GRPEL2 GrpE protein homolog 2, mitochondrial
DIAPH1 Protein diaphanous homolog 1
FAM114A1 Protein NOXP20
PAPSS1 Bifunctional 3'-phosphoadenosine 5'-phosphosulfate synthase 1
FBXL18 F-box/LRR-repeat protein 18
DNAJA2 DnaJ homolog subfamily A member 2
GLMN Glomulin
NFATC2IP NFATC2-interacting protein
INPPL1 Phosphatidylinositol 3,4,5-trisphosphate 5-phosphatase 2
LXN Latexin
GAPVD1 GTPase-activating protein and VPS9 domain-containing protein 1
ARHGEF1 Rho guanine nucleotide exchange factor 1
GAMT Guanidinoacetate N-methyltransferase
CIAPIN1 Anamorsin
GCLM Glutamate--cysteine ligase regulatory subunit
SCYL1 N-terminal kinase-like protein
DYNLT1 Dynein light chain Tctex-type 1
AP3S2 AP-3 complex subunit sigma-2
UBLCP1 Ubiquitin-like domain-containing CTD phosphatase 1
ZNF503 Zinc finger protein 503
PPP6R1 Serine/threonine-protein phosphatase 6 regulatory subunit 1
CDK6 Cyclin-dependent kinase 6
C1QL1 C1q-related factor
PRPS2 Ribose-phosphate pyrophosphokinase 2
LPIN2 Phosphatidate phosphatase LPIN2
REL Proto-oncogene c-Rel
GNB1 Guanine nucleotide-binding protein G(I)/G(S)/G(T) subunit beta-1
MICU2 Calcium uptake protein 2, mitochondrial
DNAJC3 DnaJ homolog subfamily C member 3
ERO1A ERO1-like protein alpha
MYDGF Myeloid-derived growth factor
KDELC2 Protein O-glucosyltransferase 3
CALU Calumenin
UGGT1 UDP-glucose:glycoprotein glucosyltransferase 1
CALR Calreticulin
SERPINH1 Serpin H1
HSP90B1 Endoplasmin
PDIA4 Protein disulfide-isomerase A4
SLC1A1 Excitatory amino acid transporter 3
CCT6B T-complex protein 1 subunit zeta-2
FKBP9 Peptidyl-prolyl cis-trans isomerase FKBP9
TSPAN15 Tetraspanin-15
AFF1 AF4/FMR2 family member 1
CES3 Carboxylesterase 3
LTF Lactotransferrin
LRPAP1 Alpha-2-macroglobulin receptor-associated protein
LOXL2 Lysyl oxidase homolog 2
ITGA3 Integrin alpha-3
ADAM9 Disintegrin and metalloproteinase domain-containing protein 9
LAMB2 Laminin subunit beta-2
LRRFIP2 Leucine-rich repeat flightless-interacting protein 2
SGMS2 Phosphatidylcholine:ceramide cholinephosphotransferase 2

Fig S1. *Cont.*

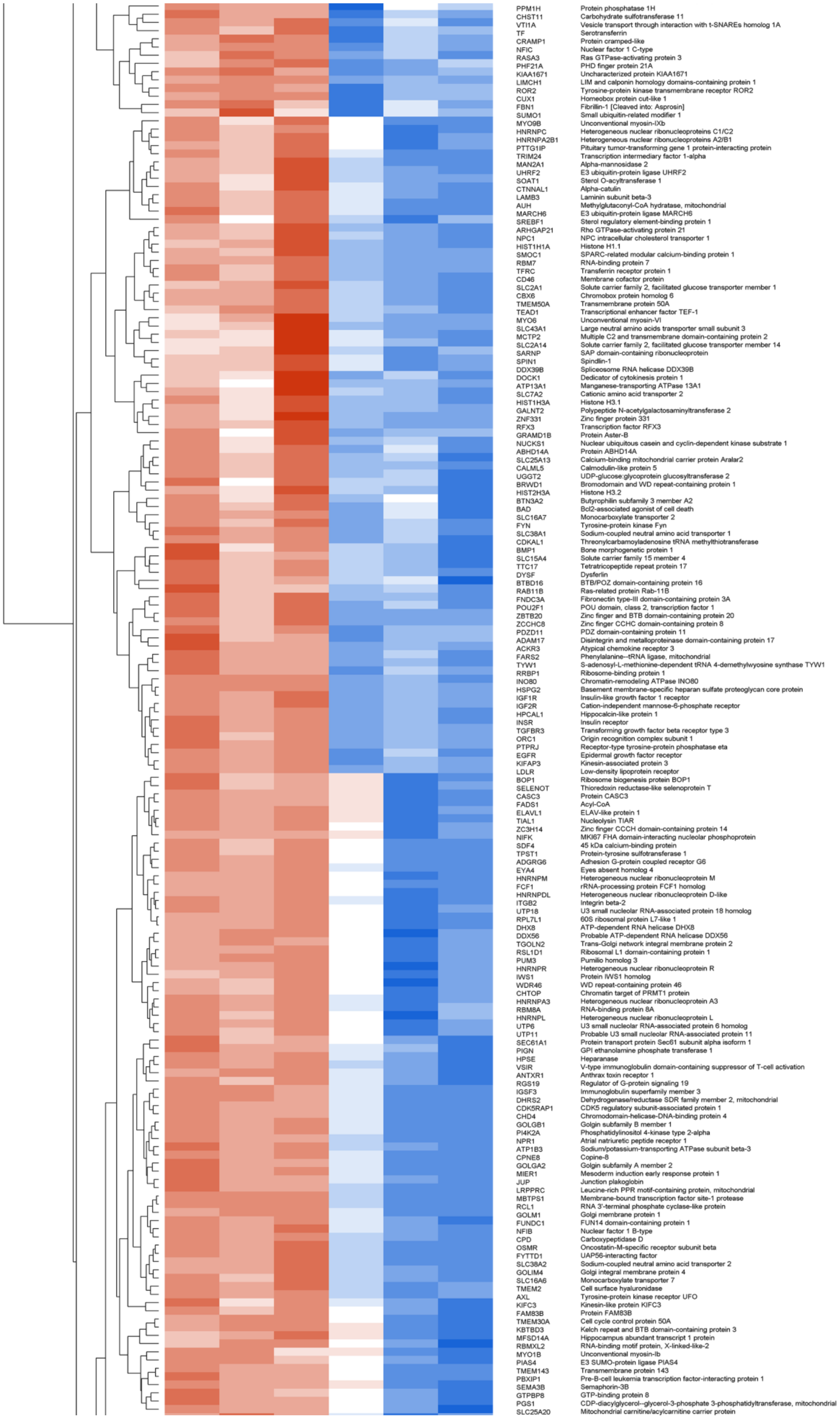
PPM1H Protein phosphatase 1H
CHST11 Carbohydrate sulfotransferase 11
VTI1A Vesicle transport through interaction with t-SNAREs homolog 1A
TF Serotransferrin
CRAMP1 Protein cramped-like
NFIC Nuclear factor 1 C-type
RASA3 Ras GTPase-activating protein 3
PHF21A PHD finger protein 21A
KIAA1671 Uncharacterized protein KIAA1671
LIMCH1 LIM and calponin homology domains-containing protein 1
ROR2 Tyrosine-protein kinase transmembrane receptor ROR2
CUX1 Homeobox protein cut-like 1
FBN1 Fibrillin-1 [Cleaved into: Asprosin]
SUMO1 Small ubiquitin-related modifier 1
MYO9B Unconventional myosin-IXb
HNRNPC Heterogeneous nuclear ribonucleoproteins C1/C2
HNRNPA2B1 Heterogeneous nuclear ribonucleoproteins A2/B1
PTTG1IP Pituitary tumor-transforming gene 1 protein-interacting protein
TRIM24 Transcription intermediary factor 1-alpha
MAN2A1 Alpha-mannosidase 2
UHRF2 E3 ubiquitin-protein ligase UHRF2
SOAT1 Sterol O-acyltransferase 1
CTNNAL1 Alpha-catulin
LAMB3 Laminin subunit beta-3
AUH Methylglutaconyl-CoA hydratase, mitochondrial
MARCH6 E3 ubiquitin-protein ligase MARCH6
SREBF1 Sterol regulatory element-binding protein 1
ARHGAP21 Rho GTPase-activating protein 21
NPC1 NPC intracellular cholesterol transporter 1
HIST1H1A Histone H1.1
SMOC1 SPARC-related modular calcium-binding protein 1
RBM7 RNA-binding protein 7
TFRC Transferrin receptor protein 1
CD46 Membrane cofactor protein
SLC2A1 Solute carrier family 2, facilitated glucose transporter member 1
CBX6 Chromobox protein homolog 6
TMEM50A Transmembrane protein 50A
TEAD1 Transcriptional enhancer factor TEF-1
MYO6 Unconventional myosin-VI
SLC43A1 Large neutral amino acids transporter small subunit 3
MCTP2 Multiple C2 and transmembrane domain-containing protein 2
SLC2A14 Solute carrier family 2, facilitated glucose transporter member 14
SARNP SAP domain-containing ribonucleoprotein
SPIN1 Spindlin-1
DDX39B Spliceosome RNA helicase DDX39B
DOCK1 Dedicator of cytokinesis protein 1
ATP13A1 Manganese-transporting ATPase 13A1
SLC7A2 Cationic amino acid transporter 2
HIST1H3A Histone H3.1
GALNT2 Polypeptide N-acetylgalactosaminyltransferase 2
ZNF331 Zinc finger protein 331
RFX3 Transcription factor RFX3
GRAMD1B Protein Aster-B
NUCKS1 Nuclear ubiquitous casein and cyclin-dependent kinase substrate 1
ABHD14A Protein ABHD14A
SLC25A13 Calcium-binding mitochondrial carrier protein Aralar2
CALML5 Calmodulin-like protein 5
UGGT2 UDP-glucose:glycoprotein glucosyltransferase 2
BRWD1 Bromodomain and WD repeat-containing protein 1
HIST2H3A Histone H3.2
BTN3A2 Butyrophilin subfamily 3 member A2
BAD Bcl2-associated agonist of cell death
SLC16A7 Monocarboxylate transporter 2
FYN Tyrosine-protein kinase Fyn
SLC38A1 Sodium-coupled neutral amino acid transporter 1
CDKAL1 Threonylcarbamoyladenosine tRNA methylthiotransferase
BMP1 Bone morphogenetic protein 1
SLC15A4 Solute carrier family 15 member 4
TTC17 Tetratricopeptide repeat protein 17
DYSF Dysferlin
BTBD16 BTB/POZ domain-containing protein 16
RAB11B Ras-related protein Rab-11B
FNDC3A Fibronectin type-III domain-containing protein 3A
POU2F1 POU domain, class 2, transcription factor 1
ZBTB20 Zinc finger and BTB domain-containing protein 20
ZCCHC8 Zinc finger CCHC domain-containing protein 8
PDZD11 PDZ domain-containing protein 11
ADAM17 Disintegrin and metalloproteinase domain-containing protein 17
ACKR3 Atypical chemokine receptor 3
FARS2 Phenylalanine--tRNA ligase, mitochondrial
TYW1 S-adenosyl-L-methionine-dependent tRNA 4-demethylwyosine synthase TYW1
RRBP1 Ribosome-binding protein 1
INO80 Chromatin-remodeling ATPase INO80
HSPG2 Basement membrane-specific heparan sulfate proteoglycan core protein
IGF1R Insulin-like growth factor 1 receptor
IGF2R Cation-independent mannose-6-phosphate receptor
HPCAL1 Hippocalcin-like protein 1
INSR Insulin receptor
TGFBR3 Transforming growth factor beta receptor type 3
ORC1 Origin recognition complex subunit 1
PTPRJ Receptor-type tyrosine-protein phosphatase eta
EGFR Epidermal growth factor receptor
KIFAP3 Kinesin-associated protein 3
LDLR Low-density lipoprotein receptor
BOP1 Ribosome biogenesis protein BOP1
SELENOT Thioredoxin reductase-like selenoprotein T
CASC3 Protein CASC3
FADS1 Acyl-CoA
ELAVL1 ELAV-like protein 1
TIAL1 Nucleolysin TIAR
ZC3H14 Zinc finger CCCH domain-containing protein 14
NIFK MKI67 FHA domain-interacting nucleolar phosphoprotein
SDF4 45 kDa calcium-binding protein
TPST1 Protein-tyrosine sulfotransferase 1
ADGRG6 Adhesion G-protein coupled receptor G6
EYA4 Eyes absent homolog 4
HNRNPM Heterogeneous nuclear ribonucleoprotein M
FCF1 rRNA-processing protein FCF1 homolog
HNRNPDL Heterogeneous nuclear ribonucleoprotein D-like
ITGB2 Integrin beta-2
UTP18 U3 small nucleolar RNA-associated protein 18 homolog
RPL7L1 60S ribosomal protein L7-like 1
DHX8 ATP-dependent RNA helicase DHX8
DDX56 Probable ATP-dependent RNA helicase DDX56
TGOLN2 Trans-Golgi network integral membrane protein 2
RSL1D1 Ribosomal L1 domain-containing protein 1
PUM3 Pumilio homolog 3
HNRNPR Heterogeneous nuclear ribonucleoprotein R
IWS1 Protein IWS1 homolog
WDR46 WD repeat-containing protein 46
CHTOP Chromatin target of PRMT1 protein
HNRNPA3 Heterogeneous nuclear ribonucleoprotein A3
RBM8A RNA-binding protein 8A
HNRNPL Heterogeneous nuclear ribonucleoprotein L
UTP6 U3 small nucleolar RNA-associated protein 6 homolog
UTP11 Probable U3 small nucleolar RNA-associated protein 11
SEC61A1 Protein transport protein Sec61 subunit alpha isoform 1
PIGN GPI ethanolamine phosphate transferase 1
HPSE Heparanase
VSIR V-type immunoglobulin domain-containing suppressor of T-cell activation
ANTXR1 Anthrax toxin receptor 1
RGS19 Regulator of G-protein signaling 19
IGSF3 Immunoglobulin superfamily member 3
DHRS2 Dehydrogenase/reductase SDR family member 2, mitochondrial
CDK5RAP1 CDK5 regulatory subunit-associated protein 1
CHD4 Chromodomain-helicase-DNA-binding protein 4
GOLGB1 Golgin subfamily B member 1
PI4K2A Phosphatidylinositol 4-kinase type 2-alpha
NPR1 Atrial natriuretic peptide receptor 1
ATP1B3 Sodium/potassium-transporting ATPase subunit beta-3
CPNE8 Copine-8
GOLGA2 Golgin subfamily A member 2
MIER1 Mesoderm induction early response protein 1
JUP Junction plakoglobin
LRPPRC Leucine-rich PPR motif-containing protein, mitochondrial
MBTPS1 Membrane-bound transcription factor site-1 protease
RCL1 RNA 3'-terminal phosphate cyclase-like protein
GOLM1 Golgi membrane protein 1
FUNDC1 FUN14 domain-containing protein 1
NFIB Nuclear factor 1 B-type
CPD Carboxypeptidase D
OSMR Oncostatin-M-specific receptor subunit beta
FYTTD1 UAP56-interacting factor
SLC38A2 Sodium-coupled neutral amino acid transporter 2
GOLIM4 Golgi integral membrane protein 4
SLC16A6 Monocarboxylate transporter 7
TMEM2 Cell surface hyaluronidase
AXL Tyrosine-protein kinase receptor UFO
KIFC3 Kinesin-like protein KIFC3
FAM83B Protein FAM83B
TMEM30A Cell cycle control protein 50A
KBTBD3 Kelch repeat and BTB domain-containing protein 3
MFSD14A Hippocampus abundant transcript 1 protein
RBMXL2 RNA-binding motif protein, X-linked-like-2
MYO1B Unconventional myosin-Ib
PIAS4 E3 SUMO-protein ligase PIAS4
TMEM143 Transmembrane protein 143
PBXIP1 Pre-B-cell leukemia transcription factor-interacting protein 1
SEMA3B Semaphorin-3B
GTPBP8 GTP-binding protein 8
PGS1 CDP-diacylglycerol--glycerol-3-phosphate 3-phosphatidyltransferase, mitochondrial
SLC25A20 Mitochondrial carnitine/acylcarnitine carrier protein

Fig S1. *Cont.*

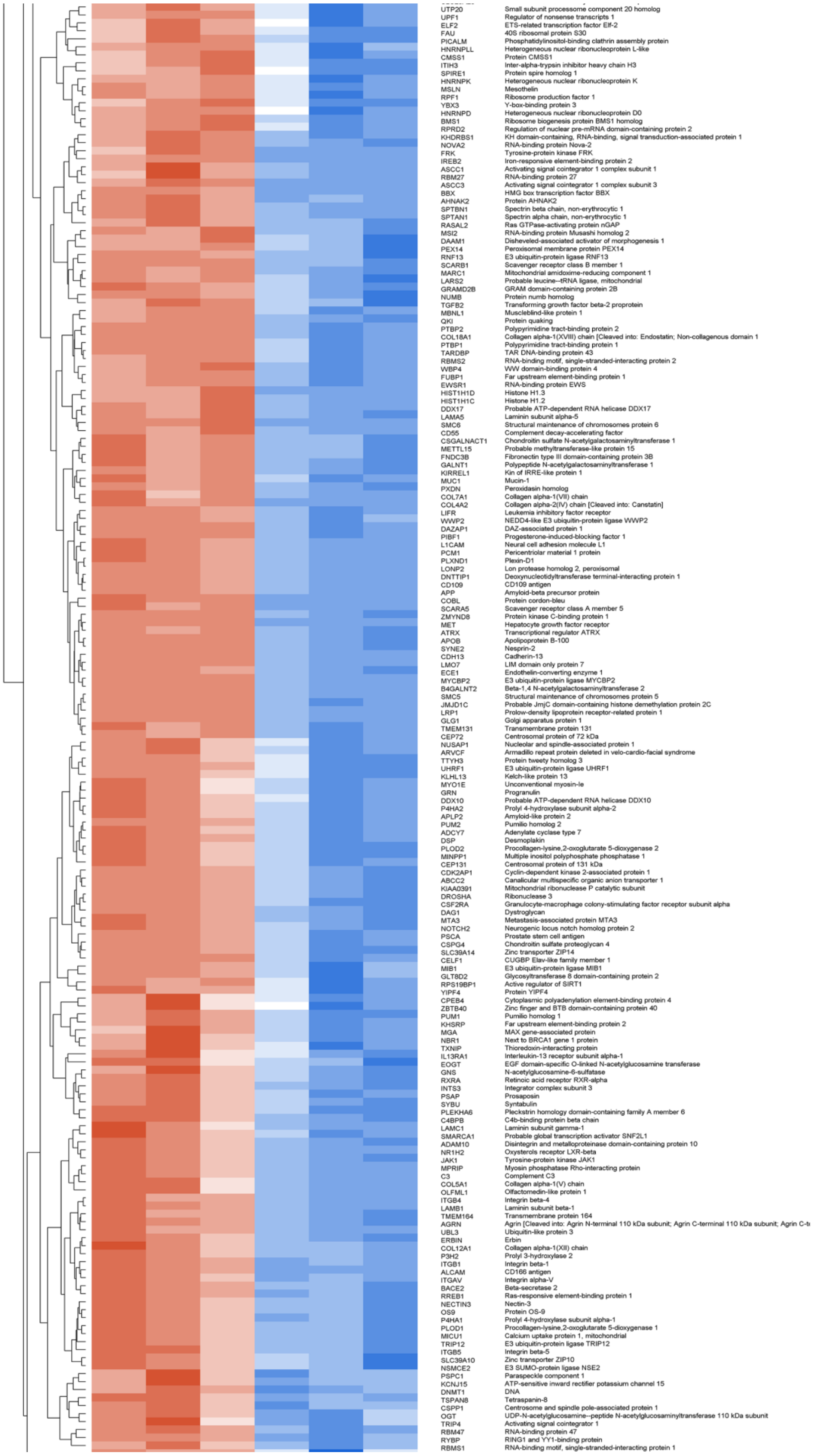
UTP20 Small subunit processome component 20 homolog
UPF1 Regulator of nonsense transcripts 1
ELF2 ETS-related transcription factor Elf-2
FAU 40S ribosomal protein S30
PICALM Phosphatidylinositol-binding clathrin assembly protein
HNRNPLL Heterogeneous nuclear ribonucleoprotein L-like
CMSS1 Protein CMSS1
ITIH3 Inter-alpha-trypsin inhibitor heavy chain H3
SPIRE1 Protein spire homolog 1
HNRNPK Heterogeneous nuclear ribonucleoprotein K
MSLN Mesothelin
RPF1 Ribosome production factor 1
YBX3 Y-box-binding protein 3
HNRNPD Heterogeneous nuclear ribonucleoprotein D0
BMS1 Ribosome biogenesis protein BMS1 homolog
RPRD2 Regulation of nuclear pre-mRNA domain-containing protein 2
KHDRBS1 KH domain-containing, RNA-binding, signal transduction-associated protein 1
NOVA2 RNA-binding protein Nova-2
FRK Tyrosine-protein kinase FRK
IREB2 Iron-responsive element-binding protein 2
ASCC1 Activating signal cointegrator 1 complex subunit 1
RBM27 RNA-binding protein 27
ASCC3 Activating signal cointegrator 1 complex subunit 3
BBX HMG box transcription factor BBX
AHNAK2 Protein AHNAK2
SPTBN1 Spectrin beta chain, non-erythrocytic 1
SPTAN1 Spectrin alpha chain, non-erythrocytic 1
RASAL2 Ras GTPase-activating protein nGAP
MSI2 RNA-binding protein Musashi homolog 2
DAAM1 Disheveled-associated activator of morphogenesis 1
PEX14 Peroxisomal membrane protein PEX14
RNF13 E3 ubiquitin-protein ligase RNF13
SCARB1 Scavenger receptor class B member 1
MARC1 Mitochondrial amidoxime-reducing component 1
LARS2 Probable leucine--tRNA ligase, mitochondrial
GRAMD2B GRAM domain-containing protein 2B
NUMB Protein numb homolog
TGFB2 Transforming growth factor beta-2 proprotein
MBNL1 Muscleblind-like protein 1
QKI Protein quaking
PTBP2 Polypyrimidine tract-binding protein 2
COL18A1 Collagen alpha-1(XVIII) chain [Cleaved into: Endostatin; Non-collagenous domain 1
PTBP1 Polypyrimidine tract-binding protein 1
TARDBP TAR DNA-binding protein 43
RBMS2 RNA-binding motif, single-stranded-interacting protein 2
WBP4 WW domain-binding protein 4
FUBP1 Far upstream element-binding protein 1
EWSR1 RNA-binding protein EWS
HIST1H1D Histone H1.3
HIST1H1C Histone H1.2
DDX17 Probable ATP-dependent RNA helicase DDX17
LAMA5 Laminin subunit alpha-5
SMC6 Structural maintenance of chromosomes protein 6
CD55 Complement decay-accelerating factor
CSGALNACT1 Chondroitin sulfate N-acetylgalactosaminyltransferase 1
METTL15 Probable methyltransferase-like protein 15
FNDC3B Fibronectin type III domain-containing protein 3B
GALNT1 Polypeptide N-acetylgalactosaminyltransferase 1
KIRREL1 Kin of IRRE-like protein 1
MUC1 Mucin-1
PXDN Peroxidasin homolog
COL7A1 Collagen alpha-1(VII) chain
COL4A2 Collagen alpha-2(IV) chain [Cleaved into: Canstatin]
LIFR Leukemia inhibitory factor receptor
WWP2 NEDD4-like E3 ubiquitin-protein ligase WWP2
DAZAP1 DAZ-associated protein 1
PIBF1 Progesterone-induced-blocking factor 1
L1CAM Neural cell adhesion molecule L1
PCM1 Pericentriolar material 1 protein
PLXND1 Plexin-D1
LONP2 Lon protease homolog 2, peroxisomal
DNTTIP1 Deoxynucleotidyltransferase terminal-interacting protein 1
CD109 CD109 antigen
APP Amyloid-beta precursor protein
COBL Protein cordon-bleu
SCARA5 Scavenger receptor class A member 5
ZMYND8 Protein kinase C-binding protein 1
MET Hepatocyte growth factor receptor
ATRX Transcriptional regulator ATRX
APOB Apolipoprotein B-100
SYNE2 Nesprin-2
CDH13 Cadherin-13
LMO7 LIM domain only protein 7
ECE1 Endothelin-converting enzyme 1
MYCBP2 E3 ubiquitin-protein ligase MYCBP2
B4GALNT2 Beta-1,4 N-acetylgalactosaminyltransferase 2
SMC5 Structural maintenance of chromosomes protein 5
JMJD1C Probable JmjC domain-containing histone demethylation protein 2C
LRP1 Prolow-density lipoprotein receptor-related protein 1
GLG1 Golgi apparatus protein 1
TMEM131 Transmembrane protein 131
CEP72 Centrosomal protein of 72 kDa
NUSAP1 Nucleolar and spindle-associated protein 1
ARVCF Armadillo repeat protein deleted in velo-cardio-facial syndrome
TTYH3 Protein tweety homolog 3
UHRF1 E3 ubiquitin-protein ligase UHRF1
KLHL13 Kelch-like protein 13
MYO1E Unconventional myosin-Ie
GRN Progranulin
DDX10 Probable ATP-dependent RNA helicase DDX10
P4HA2 Prolyl 4-hydroxylase subunit alpha-2
APLP2 Amyloid-like protein 2
PUM2 Pumilio homolog 2
ADCY7 Adenylate cyclase type 7
DSP Desmoplakin
PLOD2 Procollagen-lysine,2-oxoglutarate 5-dioxygenase 2
MINPP1 Multiple inositol polyphosphate phosphatase 1
CEP131 Centrosomal protein of 131 kDa
CDK2AP1 Cyclin-dependent kinase 2-associated protein 1
ABCC2 Canalicular multispecific organic anion transporter 1
KIAA0391 Mitochondrial ribonuclease P catalytic subunit
DROSHA Ribonuclease 3
CSF2RA Granulocyte-macrophage colony-stimulating factor receptor subunit alpha
DAG1 Dystroglycan
MTA3 Metastasis-associated protein MTA3
NOTCH2 Neurogenic locus notch homolog protein 2
PSCA Prostate stem cell antigen
CSPG4 Chondroitin sulfate proteoglycan 4
SLC39A14 Zinc transporter ZIP14
CELF1 CUGBP Elav-like family member 1
MIB1 E3 ubiquitin-protein ligase MIB1
GLT8D2 Glycosyltransferase 8 domain-containing protein 2
RPS19BP1 Active regulator of SIRT1
YIPF4 Protein YIPF4
CPEB4 Cytoplasmic polyadenylation element-binding protein 4
ZBTB40 Zinc finger and BTB domain-containing protein 40
PUM1 Pumilio homolog 1
KHSRP Far upstream element-binding protein 2
MGA MAX gene-associated protein
NBR1 Next to BRCA1 gene 1 protein
TXNIP Thioredoxin-interacting protein
IL13RA1 Interleukin-13 receptor subunit alpha-1
EOGT EGF domain-specific O-linked N-acetylglucosamine transferase
GNS N-acetylglucosamine-6-sulfatase
RXRA Retinoic acid receptor RXR-alpha
INTS3 Integrator complex subunit 3
PSAP Prosaposin
SYBU Syntabulin
PLEKHA6 Pleckstrin homology domain-containing family A member 6
C4BPB C4b-binding protein beta chain
LAMC1 Laminin subunit gamma-1
SMARCA1 Probable global transcription activator SNF2L1
ADAM10 Disintegrin and metalloproteinase domain-containing protein 10
NR1H2 Oxysterols receptor LXR-beta
JAK1 Tyrosine-protein kinase JAK1
MPRIP Myosin phosphatase Rho-interacting protein
C3 Complement C3
COL5A1 Collagen alpha-1(V) chain
OLFML1 Olfactomedin-like protein 1
ITGB4 Integrin beta-4
LAMB1 Laminin subunit beta-1
TMEM164 Transmembrane protein 164
AGRN Agrin [Cleaved into: Agrin N-terminal 110 kDa subunit; Agrin C-terminal 110 kDa subunit; Agrin C-t
UBL3 Ubiquitin-like protein 3
ERBIN Erbin
COL12A1 Collagen alpha-1(XII) chain
P3H2 Prolyl 3-hydroxylase 2
ITGB1 Integrin beta-1
ALCAM CD166 antigen
ITGAV Integrin alpha-V
BACE2 Beta-secretase 2
RREB1 Ras-responsive element-binding protein 1
NECTIN3 Nectin-3
OS9 Protein OS-9
P4HA1 Prolyl 4-hydroxylase subunit alpha-1
PLOD1 Procollagen-lysine,2-oxoglutarate 5-dioxygenase 1
MICU1 Calcium uptake protein 1, mitochondrial
TRIP12 E3 ubiquitin-protein ligase TRIP12
ITGB5 Integrin beta-5
SLC39A10 Zinc transporter ZIP10
NSMCE2 E3 SUMO-protein ligase NSE2
PSPC1 Paraspeckle component 1
KCNJ15 ATP-sensitive inward rectifier potassium channel 15
DNMT1 DNA
TSPAN8 Tetraspanin-8
CSPP1 Centrosome and spindle pole-associated protein 1
OGT UDP-N-acetylglucosamine--peptide N-acetylglucosaminyltransferase 110 kDa subunit
TRIP4 Activating signal cointegrator 1
RBM47 RNA-binding protein 47
RYBP RING1 and YY1-binding protein
RBMS1 RNA-binding motif, single-stranded-interacting protein 1

Fig S1. *Cont.*

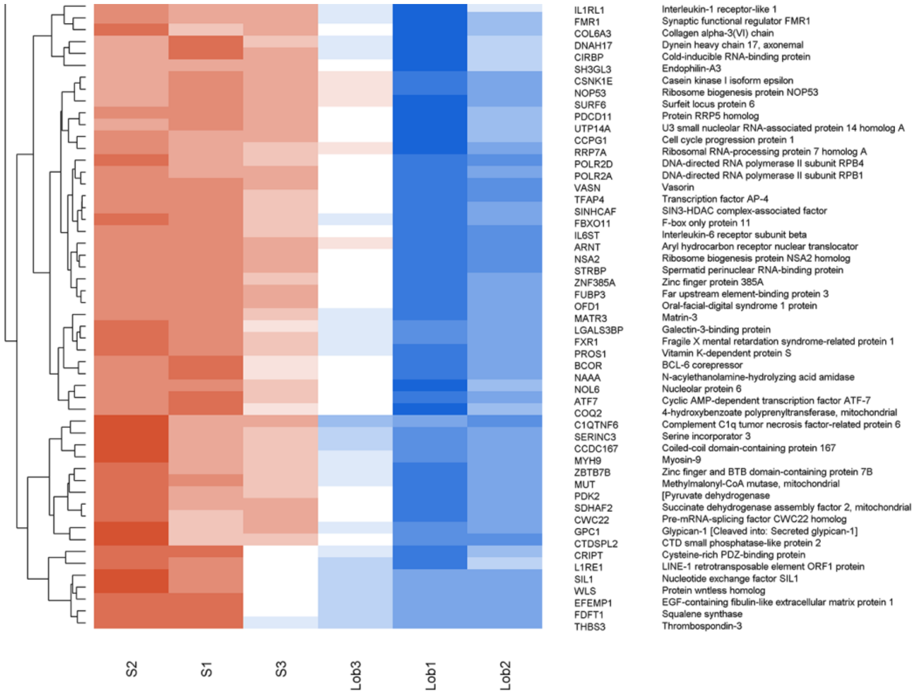

Supplementary Figure 1. Hierarchical clustering of differentially expressed proteins in SaOS-2 osteosarcoma cells between the CG and LG. Each group contains three samples. A total of 1,251 proteins that changed significantly, including 819 proteins upregulated and 432 proteins downregulated ($p < 0.05$), were identified.

2. Supplementary Tables

Table S1. Cluster analysis of significantly changed proteins between LG and rhILG based on STEM

Accession Number	Protein Names （Gene Names）	Cluster	P-value
Q9P021	Cysteine-rich PDZ-binding protein(CRIPT)	2	0.02624
P51114	Fragile X mental retardation syndrome-related protein 1(FXR1)	2	0.01453
Q92945	Far upstream element-binding protein 2(KHSRP)	2	0.01643
Q13283	Ras GTPase-activating protein-binding protein 1(G3BP1)	2	0.01424
Q9UFH2	Dynein heavy chain 17(DNAH17)	2	0.04828
P16989	Y-box-binding protein 3(YBX3)	2	0.009454
Q96AE4	Far upstream element-binding protein 1(FUBP1)	2	0.034561
Q17RY0	Cytoplasmic polyadenylation element-binding protein 4(CPEB4)	2	0.005776
Q8IWE2	Protein NOXP20(FAM114A1)	5	0.006561
P54851	Epithelial membrane protein 2(EMP2)	5	0.01223
Q01995	Transgelin(TAGLN)	5	0.028617
O43175	D-3-phosphoglycerate dehydrogenase(PHGDH)	5	0.000678
P56277	Cx9C motif-containing protein 4(CMC4)	5	0.045793
Q15126	Phosphomevalonate kinase (PMVK)	5	0.005733
Q9BXW6	Oxysterol-binding protein-related protein 1(OSBPL1A)	5	0.011028
O95671	N-acetylserotonin O-methyltransferase-like protein (ASMTL)	5	0.003861
A6NDU8	UPF0600 protein C5orf51(C5orf51)	5	0.04245
Q8WUH6	Transmembrane protein 263 (TMEM263)	5	0.040527

Q9BZV1	UBX domain-containing protein 6 (UBXN6)	5	0.009873
Q8N5M9	Protein jagunal homolog 1(JAGN1)	5	0.033991
Q9UHR6	Zinc finger HIT domain-containing protein 2 (ZNHIT2)	5	0.008993
Q8N4V1	Membrane magnesium transporter 1 (MMGT1)	5	0.037916

Table S2. Parallel reaction monitoring validation of proteins with significant difference in TMT labeling LC-MS/MS

Protein Name	Protein Accessions	Gene Name	Ratio of rhILG/LG with PRM	P-value of PRM	Ratio of rhILG/LG with TMT	P-value of TMT
Fragile X mental retardation syndrome-related protein 1	P51114	FXR1	1.63	0.0002	1.23	0.0145
Far upstream element-binding protein 2	Q92945	KHSRP	1.68	0.0017	1.24	0.0164
Ras GTPase-activating protein-binding protein 1	Q13283	G3BP1	1.51	0.0027	1.25	0.0142
Y-box-binding protein 3	P16989	YBX3	1.62	0.0038	1.27	0.0095
Far upstream element-binding protein 1	Q96AE4	FUBP1	1.68	0.0045	1.28	0.0346
Protein NOXP20	Q8IWE2	FAM114A1	0.7	0.0061	0.78	0.0066
Transgelin	Q01995	TAGLN	0.74	0.0064	0.79	0.0286
D-3-phosphoglycerate dehydrogenase	O43175	PHGDH	0.69	0.0075	0.79	0.0007
Phosphomevalonate kinase	Q15126	PMVK	0.47	0.0093	0.81	0.0057
Protein jagunal homolog 1	Q8N5M9	JAGN1	0.79	0.0144	0.83	0.0340
Proto-oncogene c-Fos	P01100	FOS	0.62	0.0154	0.74	0.0277
Keratin, type I cytoskeletal 17	Q04695	KRT17	0.74	0.0247	0.82	0.0066
Keratin, type II cytoskeletal 7	P08729	KRT7	0.81	0.0249	0.83	0.0233
Adenylate kinase 2, mitochondrial	P54819	AK2	0.75	0.02534	0.83	0.0451
Cytochrome c	P99999	CYCS	0.72	0.0256	0.8	0.0377
Gamma-interferon-inducible protein 16	Q16666	IFI16	1.54	0.02674	1.23	0.0212

Protein Name	Protein Accessions	Gene Name	Ratio of rhILG/LG with PRM	P value of PRM	Ratio of rhILG/LG with TMT	P value of TMT
Caprin-1	Q14444	CAPRIN1	1.53	0.0277	1.28	0.0479
Inositol 1,4,5-trisphosphate receptor type 1	Q14643	ITPR1	0.72	0.0356	0.83	0.0079
Ras GTPase-activating protein-binding protein 2	Q9UN86	G3BP2	1.49	0.0366	1.29	0.0296
Protein regulator of cytokinesis 1	O43663	PRC1	1.58	0.0385	1.23	0.0323
Ubiquitin-associated protein 2-like	Q14157	UBAP2L	1.55	0.0402	1.25	0.0125
Hexokinase-2	P52789	HK2	0.58	0.0427	0.79	0.0324
Cold shock domain-containing protein E1	O75534	CSDE1	1.73	0.0524	1.21	0.0294
Rac GTPase-activating protein 1	Q9H0H5	RACGAP1	1.32	0.0576	1.21	0.0132
Kinesin-like protein KIF2C	Q99661	KIF2C	1.76	0.0578	1.3	0.0250
Anillin	Q9NQW6	ANLN	1.67	0.0592	1.21	0.0336
PCNA-associated factor	Q15004	PCLAF	1.47	0.0599	1.31	0.0493
Kinesin-like protein KIF18B	Q86Y91	KIF18B	2.08	0.0888	1.31	0.0485
E3 ubiquitin-protein ligase synoviolin	Q86TM6	SYVN1	0.78	0.1229	0.78	0.0371
Mediator of RNA polymerase II transcription subunit 23	Q9ULK4	MED23	1.27	0.2406	1.22	0.0366
CCR4-NOT transcription complex subunit 10	Q9H9A5	CNOT10	1.24	0.2886	1.2	0.0441

Table S3. Clinical characteristics of osteosarcoma patients with platinum-based chemotherapy

Gender	Age	Clinic Diagnosis	Pathological Diagnosis	Staging	Response to Platinum	Distant Metastasis	Die or Survival
Female	19	Right distal femur OS	Common type OS	IIB	Sensitive	No	Survival
Female	32	Right distal femur tumor	Parosteal OS	IIB	Sensitive	No	Survival
Male	22	Left distal femur OS	Talangiectatic OS	IIB	Sensitive	No	Survival with amputation
Female	37	Left femur OS combined with pathological fracture	Fibroblastic OS	IIB	Sensitive	No	Survival with amputation
Female	15	Left upper tibia OS	Common type OS	IIA	Sensitive	No	Survival
Male	18	Left proximal tibia mass	Common type OS	IIA	Sensitive	No	Survival
Male	15	Right proximal tibia tumor	Multiple or metastatic OS	III	Resistant	Pelvic metastasis	Die
Male	15	Right distal femur mass	Small cell OS	III	Resistant	Pulmonary metastasis	Survival
Male	20	Distal femur tumor	Fibroblastic OS	IIB	Resistant	No	Die
Male	13	Right proximal femur mass	common type OS	III	Resistant	Pulmonary metastasis	Die
Male	19	Right pelvic OS	Small cell OS	III	Resistant	Pulmonary metastasis	Die

Gender	Age	Clinic Diagnosis	Pathological Diagnosis	Staging	Response to Platinum	Distant Metastasis	Die or Survival
Female	13	Right proximal humerus OS	common type with tumor thrombosis	III	Resistant	Pulmonary metastasis	Die
Male	40	Left distal femur mass	Talangiectatic OS	IIA	Resistant	No	Die
female	14	Left distal femur OS	Common type OS	III	Resistant	Pulmonary metastasis	Die
Male	18	Right distal femur mass	Fibroblastic OS	IIB	Resistant	No	Die
Male	11	Lleft distal femur OS	Common type OS	IIB	Resistant	No	Die
Male	22	Right distal femur mass	Fibroblastic OS	IIB	Sensitive	No	Survival
Male	9	Left distal femur mass	Common type OS	IIB	Sensitive	No	Survival
female	12	Left proximal tibia mass	Common type OS	IIB	Sensitive	No	Survival
female	14	Right distal femur mass	Small cell OS	IIB	Sensitive	No	Survival
female	12	Right distal femur OS	Common type OS	IIB	Sensitive	No	Survival with amputation
male	12	Left distal femur tumor	Common type OS	IIB	Resistant	No	Die
male	19	Left distal femur OS	Common type OS	IIB	Sensitive	No	Survival
female	8	Left distal femur OS	Talangiectatic OS	III	Resistant	Subcutaneous metastasis	Die
female	14	Left distal femur mass	Common type OS	IIB	Sensitive	No	Survival

Gender	Age	Clinic Diagnosis	Pathological Diagnosis	Staging	Response to Platinum	Distant Metastasis	Die or Survival
male	17	Right proximal tibia OS	Common type OS	IIA	Sensitive	No	Survival
female	14	Left femur OS	Common type OS	III	Resistant	Left humeral metastasis	Die
female	8	Left distal femur OS	Talangiectatic OS	III	Resistant	Subcutaneous metastasis	Die
female	25	Tumor outside of right calf	Common type OS	III	Resistant	Pulmonary metastasis	Die
female	16	Left proximal tibia OS	Common type OS	IIA	Sensitive	No	Survival

OS: osteosarcoma
